# Novel Hybrids of Podophyllotoxin and Coumarin Inhibit the Growth and Migration of Human Oral Squamous Carcinoma Cells

**DOI:** 10.3389/fchem.2020.626075

**Published:** 2021-01-15

**Authors:** Guohui Bai, Dan Zhao, Xin Ran, Lei Zhang, Degang Zhao

**Affiliations:** ^1^The Key Laboratory of Plant Resources Conservation and Germplasm Innovation in Mountainous Region (Ministry of Education), Institute of Agro-Bioengineering and College of Life Sciences, Guizhou University, Guiyang, China; ^2^Key Laboratory of Biocatalysis & Chiral Drug Synthesis of Guizhou Province and School of Pharmacy, Zunyi Medical University, Zunyi, China; ^3^Institute of Guizhou Distinctive Plant Resources Conservation, Guizhou Academy of Agricultural Science, Guiyang, China

**Keywords:** podophyllotoxin, coumarin, hybrid strategy, human oral squamous carcinoma cells, anticancer, molecular mechanism

## Abstract

Oral squamous cell carcinoma is the most common malignancy of oral tumor. In this study, two novel hybrids of podophyllotoxin and coumarin were designed using molecular hybridization strategy and synthesized. Pharmacological evaluation showed that the potent compound **12b** inhibited the proliferation of three human oral squamous carcinoma cell lines with nanomolar IC_50_ values, as well as displayed less toxicity on normal cells. Mechanistic studies indicated that **12b** triggered HSC-2 cell apoptosis, induced cell cycle arrest, and inhibited cell migration. Moreover, **12b** could disturb the microtubule network via binding into the tubulin. It was noteworthy that induction of autophagy by **12b** was associated with the upregulation of Beclin1, as well as LC3-II. Furthermore, **12b** significantly stimulated the AMPK pathway and restrained the AKT/mTOR pathway in HSC-2 cells. These results indicated that compound **12b** was a promising candidate for further investigation.

## Introduction

Oral squamous cell carcinoma (OSCC) is the most common malignancy of oral tumor (about 90%) worldwide (Kademani, 2007). Despite increasing development in the treatment of OSCC with surgery, radiation, and chemotherapy, only 50–60% of diagnosed patients could survive in 5 years after the initial diagnosis (Nör and Gutkind, 2018). Moreover, chemotherapeutic agents, such as docetaxel, fluorouracil, and cisplatin, have attracted great attention for their benefits in the clinical therapy of OSCC, whereas they have limited efficacy because of toxic side effects and multidrug resistance (Vogel et al., 2010). Accordingly, the development of novel anti-oral cancer candidates with less toxicity is an urgent need for OSCC patients.

**Figure 1:**
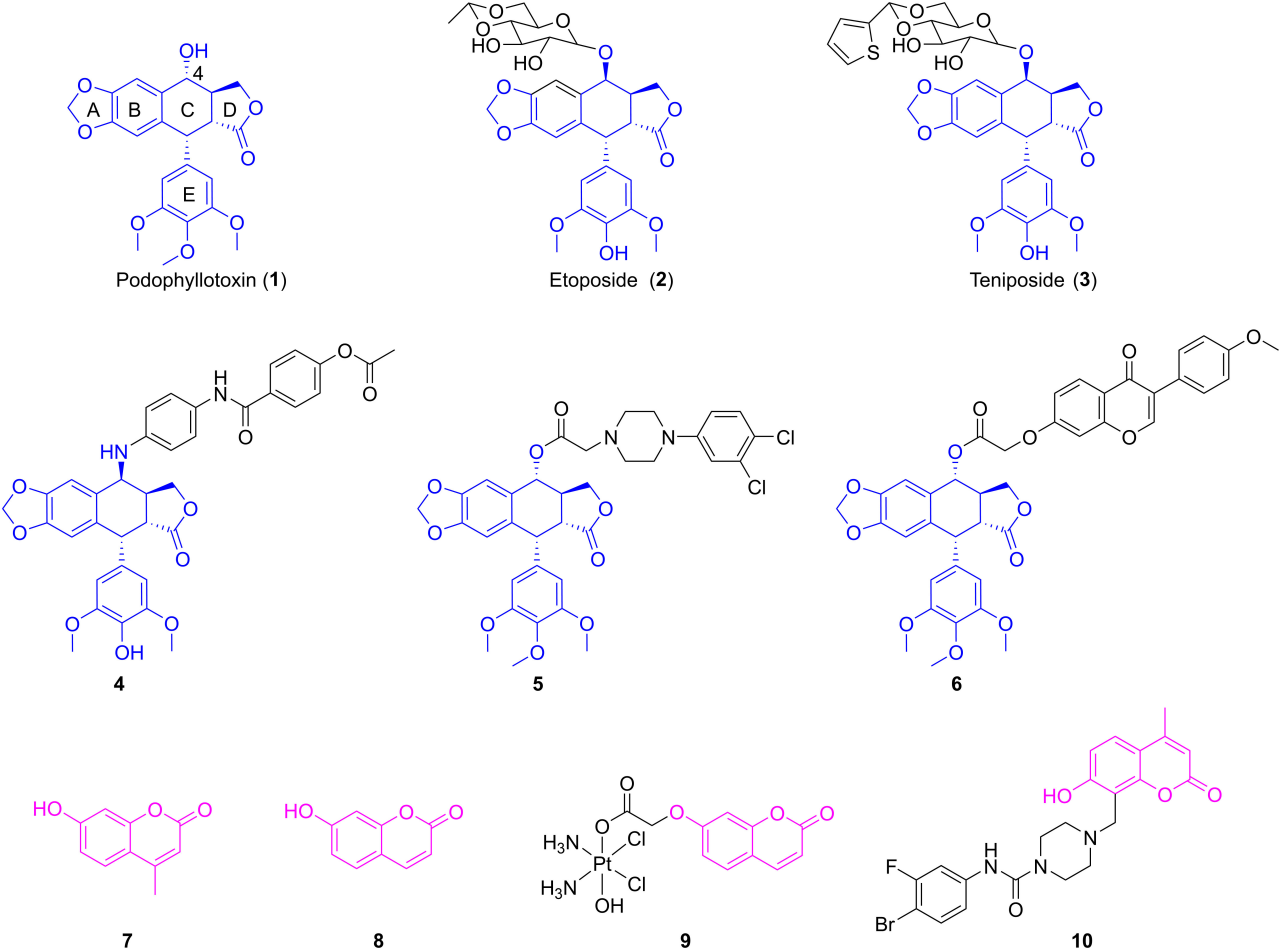
The structures of podophyllotoxin, coumarin, and their analogs.

Podophyllotoxin (**1**, Figure 1), a well-known cyclolignan derived from the roots and rhizomes of the *Podophyllum* species, is a chemotherapeutic agent with potent cytotoxic effects on various cancer cell lines via inhibition of the polymerization of tubulin (Yu et al., 2017). However, serious toxic side effects have hindered its clinical use (Bohlin and Rosen, 1996). Therefore, podophyllotoxin had become an attractive molecule for the development of anticancer drugs by structural modifications (Xiao et al., 2020). Among them, two glucosidic derivatives of podophyllotoxin, etoposide (**2**, Figure 1) and teniposide (**3**, Figure 1), show important anticancer activity by inhibition of topoisomerase II and are widely used in the chemotherapy of various types of cancer (Liu et al., 2015). However, drug resistance and several toxic side effects still hampered their clinical use. Therefore, extensive efforts have been conducted to develop novel derivatives of podophyllotoxin with improved cytotoxicity, such as NK-611, GL-331, and NPF (Kamal et al., 2015). Molecular docking simulations and structure–activity relationships of podophyllotoxin derivatives in previous works showed that the C-4 position in the skeleton of podophyllotoxin was able to accommodate a variety of structural diversifications (Zhang et al., 2018). In recent years, plenty of C-4-modified podophyllotoxin derivatives had been prepared and screened as potent antiproliferative agents against many different types of neoplasm, such as OSCC, breast cancer, and lung cancer. For example, Hu and co-workers had previously reported the synthesis of novel 4β-anilino-4′-*O*-demethyl-4-desoxypodophyllotoxin derivatives. Biological evaluation exhibited that compound **4** (Figure 1) had potent cytotoxic activity against human OSCC KB and drug-resistant KBvin cell lines (Wang et al., 2011). A series of piperazine acetate podophyllotoxin ester derivatives were synthesized by the Wang group. Further studies showed that compound **5** (Figure 1) containing chlorine groups on phenyl ring displayed high selectively inhibitory activity against human breast MCF-7 cells with an IC_50_ value of 2.78 ± 0.15 μM *in vitro* (Sun et al., 2017). Very recently, our group have reported that the hybrid of podophyllotoxin and formononetin (**6**, Figure 1) exhibited an excellent IC_50_ value of 0.753 ± 0.173 μM against human non-small-cell lung carcinoma A549 cells (Yang et al., 2019).

Coumarins (benzopyran-2-ones) are well-known compounds and have gained great attention worldwide. Many natural and synthetic molecules, such as warfarin and esculin, are composed of coumarins and exhibit significant pharmacological activities, such as anti-inflammatory, anticancer, and antioxidant activities (Fylaktakidou et al., 2004; Kostova et al., 2011; Nasr et al., 2014). Interestingly, the Wu group firstly found that 4-methylumbelliferone (**7**, Figure 1), an antispasmodic in clinical practice, was a chemical constituent in the roots of *Eucommia ulmoides* Oliv (Ji et al., 2017). Besides, 7-hydroxycoumarin (**8**, Figure 1) was isolated by Hao et al. from the leaves of *E. ulmoides* Oilv for the first time (Fen et al., 2014; Wang et al., 2019). It was reported that 4-methylumbelliferone could enhance the anticancer activity of gemcitabine against human pancreatic cancer KP1-NL cells, indicating that 4-methylumbelliferone may be a potential chemosensitizer for the combination of anticancer drug (Nakazawa et al., 2006). Recently, the Hakamada group found that 4-methylumbelliferone also had potential anticancer properties in pancreatic cancer cells *in vitro* and *in vivo* (Nagase et al., 2017). Gou et al. reported the synthesis of Cou-platin (**9**, Figure 1), which was composed of 7-hydroxycoumarin and a platinum(IV) moiety. Significantly, compound **9** exhibited potent an antitumor effect on several cancer cell lines *in vitro* and showed less toxicity. This study showed that 7-hydroxycoumarin had the potential to increase the anticancer activity of cisplatin, as well as reduce the toxicity of cisplatin (Hua et al., 2018). In addition, compound **10** (Figure 1), a derivative of 4-methylumbelliferone, exerted a significant inhibitory effect on MGC-803 and NCI-H460 cells *in vitro* with IC_50_ values of 2.13 ± 0.75 and 1.86 ± 0.73 μM, respectively (Huang et al., 2017).

**Figure 2:**
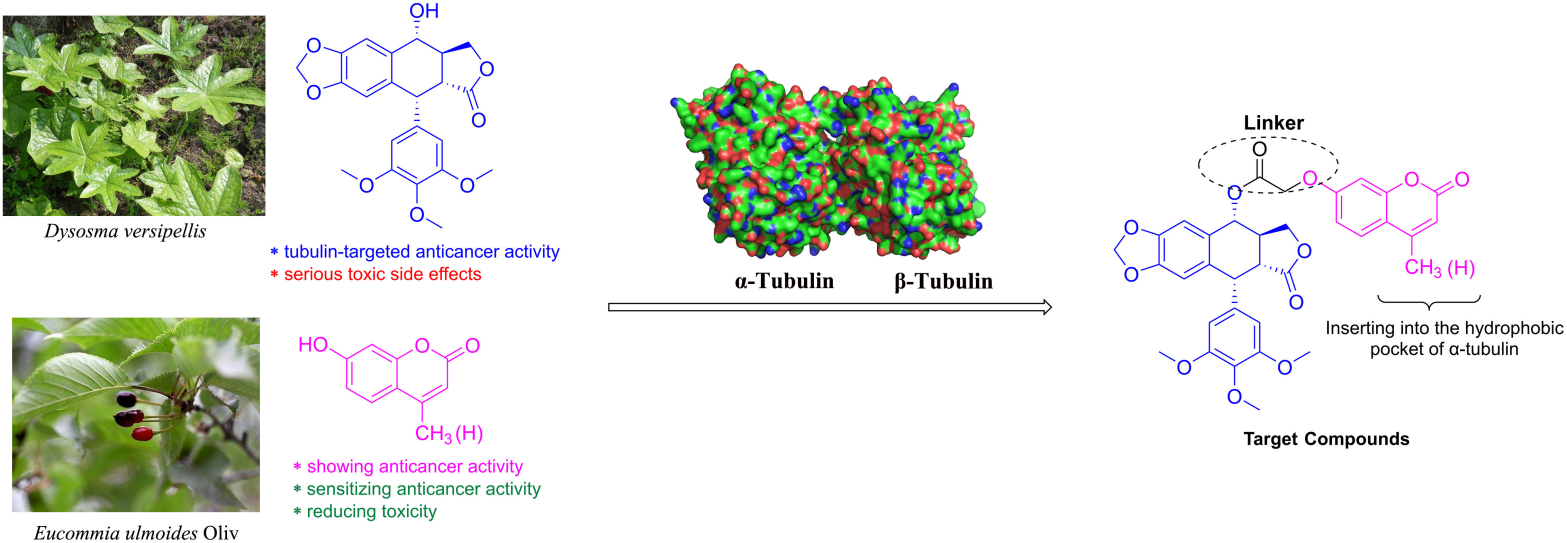
Design of the target compounds.

Molecular hybridization is an attractive strategy to design new molecules by using structural modification based on the hybridization of two or more pharmacophores or bioactive compounds into a single molecule (Mishra and Singh, 2016). Compared with the parent compounds, the target molecules designed by molecular hybridization may possess improved activity or safer toxicity profile (Hampannavar et al., 2016). The natural product podophyllotoxin is an attractive tubulin-targeted molecule for the development of anticancer drugs; however, it has serious toxic side effects. In addition, two coumarins, 4-methylumbelliferone and 7-hydroxycoumarin, were isolated from *E. ulmoides* Oilv and were reported to have some important properties, such as anticancer and sensitizing anticancer activities or reducing toxicity. Recently, Hao et al. had prepared the conjugates of 4′-demethylepipodophyllotoxin and coumarin using the click reaction. And the conjugates showed equivalent cytotoxic activities compared with those of etoposide (Hao et al., 2019). Therefore, we designed novel hybrids of podophyllotoxin and coumarin in one molecule (Figure 2) using molecular hybridization strategy, and the coumarin group might reduce the toxicity of the whole molecule, as well as insert into the hydrophobic pocket of α-tubulin. To investigate the hypothesis, in this study, hybrids of podophyllotoxin and coumarin have been synthesized and detected for the anticancer activity against OSCC cells *in vitro*. Furthermore, the mechanisms of the target hybrid were also investigated.

## Materials and Methods

### Chemistry

All analytical grade materials and reagents were purchased from commercial suppliers. ^1^H and ^13^C NMR analyses were determined using 400-MHz Agilent DD2400-MR for **11** (Palo Alto, CA) and 400-MHz Bruker Avance Neo for **12a–b** (Billerica, MA) and in the solvent of DMSO-*d*_6_: the values of the coupling constants (*J*) were recorded in Hz and the chemical shifts (δ) in ppm. High-resolution mass spectrometry–electrospray ionization (HRMS-ESI) was performed on Waters Xevo G2-S QTOF (Milford, MA). Purity was tested by Waters e2695 system (Milford, MA) using a fixed wavelength UV detector (254 nm).

### Experimental Section

#### Preparation of Intermediate 11

Podophyllotoxin (**1**, 0.7 mmol) was added to a solution of triethylamine (14 mmol) in dichloromethane (10 ml), and then chloroacetyl chloride (7 mmol) in dichloromethane (4 ml) was added dropwise to the solution. The reaction solution was stirred at room temperature for 2 h, quenched by ammonium chloride solution, and extracted with dichloromethane. The organic layer was washed with water, dried over Na_2_SO_4_, filtered, and concentrated by evaporation *in vacuo* to give the crude intermediate, which was further purified via column chromatography (DCM/MeOH = 100:1) to give the key intermediate **11**.

**11**: brown solid, yield 89%, ^1^H NMR (400 MHz, DMSO-*d*_6_) δ 7.02 (s, 1H, Ar–H), 6.56 (s, 1H, Ar–H), 6.28 (s, 2H, Ar–H), 5.99 (d, *J* = 6.4 Hz, 2H, O–CH_2_-O), 5.91 (d, *J* = 9.2 Hz, 1H, CH–Ar), 4.54 (d, *J* = 14.8 Hz, 1H, CH_2_-Cl), 4.53 (d, *J* = 4.4 Hz, 1H, CH–Ar), 4.45 (d, *J* = 15.2 Hz, 1H, CH_2_-Cl), 4.31 (t, *J* = 7.2 Hz, 1H, CH–*CH*_2_–O), 4.16 (t, *J* = 8.8 Hz, 1H, CH–*CH*_2_–O), 3.60 (s, 6H, 3′,5′-OCH_3_), 3.57 (s, 3H, 4′-OCH_3_), 3.37 (dd, *J* = 4.8 Hz, 1H, *CH*–CH_2_-O), 3.76–3.66 (m, 1H, O-C–CH); ^13^C NMR (100 MHz, DMSO-*d*_6_) δ 174.24, 168.39, 152.45, 147.87, 147.33, 136.73, 135.94, 132.78, 128.51, 109.66, 108.30, 107.76, 101.85, 74.77, 71.07, 60.32, 56.10, 44.46, 43.17, 41.95, 38.61.

#### General Preparation of Compounds 12a–b

To a solution of intermediate (**11**, 0.2 mmol) and coumarin (**7** or **8**, 0.2 mmol) in acetonitrile (3 ml), catalytic amount potassium iodide and cesium carbonate (0.2 mmol) were added. After reflux for 4 h, the mixture was filtered and washed with dichloromethane. Then, the solution was removed by evaporation *in vacuo*, and the crude product was purified by flash column chromatography (DCM/MeOH = 200:1) to yield the target compound.

**12a**: white solid, yield 48%, ^1^H NMR (400 MHz, DMSO-*d*_6_) δ 7.70 (d, *J* = 8.8 Hz, 1H, Ar–H), 6.98 (d, *J* = 2.4 Hz, 1H, Ar–H), 6.96 (dd, *J* = 8.8, 2.4 Hz, 1H, Ar–H), 6.87 (s, 1H, Ar–H), 6.56 (s, 2H, Ar–H), 6.52 (s, 1H, Ar–H), 6.25 (d, *J* = 1.2 Hz, 1H, Ar–H), 5.99 (d, *J* = 7.6 Hz, 2H, O–CH_2_-O), 5.80 (d, *J* = 5.6 Hz, 1H, CH–Ar), 4.94 (dd, *J* = 29.6, 16.4 Hz, 2H, O-C–CH_2_-O), 4.46 (dd, *J* = 9.6, 6.8 Hz, 1H, CH–Ar), 4.34 (dd, *J* = 9.6, 2.4 Hz, 1H, CH–*CH*_2_–O), 4.30 (d, *J* = 4.8 Hz, 1H, CH–*CH*_2_–O), 3.74 (s, 6H, 3′,5′-OCH_3_), 3.64 (s, 3H, 4′-OCH_3_), 3.63–3.59 (m, 1H, *CH*–CH_2_-O), 3.10–3.04 (m, 1H, O-C–CH), 2.41 (s, 3H, Ar–CH_3_); ^13^C NMR (100 MHz, DMSO-*d*_6_) δ 177.70, 168.59, 160.92, 160.54, 155.00, 153.82, 153.22, 147.94, 146.74, 138.77, 136.57, 132.83, 127.05, 114.27, 112.69, 112.03, 109.26, 108.14, 106.20, 102.08, 73.48, 70.66, 65.29, 60.42, 56.38, 55.35, 44.06, 43.80, 18.58; HRMS-ESI (*m*/*z*): calcd for C_34_H_30_O_12_Na [M+Na]^+^ 653.1635, found 653.1638. High-performance liquid chromatography (HPLC), 99.49%.

**12b**: white solid, yield 50%, ^1^H NMR (400 MHz, DMSO-*d*_6_) δ 8.01 (d, *J* = 9.6 Hz, 1H, Ar–H), 7.65 (d, *J* = 8.8 Hz, 1H, Ar–H), 6.99 (d, *J* = 2.4 Hz, 1H, Ar–H), 6.95 (dd, *J* = 8.8, 2.4 Hz, 1H, Ar–H), 6.88 (s, 1H, Ar–H), 6.56 (s, 2H, Ar–H), 6.52 (s, 1H, Ar–H), 6.34 (d, *J* = 9.6 Hz, 1H, Ar–H), 5.99 (d, *J* = 6.8 Hz, 2H, O–CH_2_-O), 5.80 (d, *J* = 6.0 Hz, 1H, CH–Ar), 4.93 (dd, *J* = 26.8, 16.4 Hz, 2H, O-C–CH_2_-O), 4.45 (dd, *J* = 9.6, 6.8 Hz, 1H, CH–Ar), 4.34 (dd, *J* = 9.6, 2.0 Hz, 1H, CH–*CH*_2_–O), 4.29 (d, *J* = 4.8 Hz, 1H, CH–*CH*_2_–O), 3.74 (s, 6H, 3′,5′-OCH_3_), 3.64 (s, 3H, 4′-OCH_3_), 6.63–6.59 (m, 1H, *CH*–CH_2_-O), 3.10–3.04 (m, 1H, O-C–CH); ^13^C NMR (100 MHz, DMSO-*d*_6_) δ 177.70, 168.57, 161.02, 160.68, 155.64, 153.22, 147.93, 146.74, 144.68, 138.77, 136.57, 132.83, 130.05, 126.88, 113.48, 113.00, 106.20, 102.06, 73.47, 70.65, 65.30, 60.42, 56.38, 44.04, 43.79; HRMS-ESI (*m*/*z*): calcd for C_33_H_28_O_12_Na [M+Na]^+^ 639.1478, found 639.1474. HPLC, 99.16%.

### Pharmacology

#### Cell Counting Kit-8 Assay *In Vitro*

The *in vitro* antiproliferative activities of target compounds were investigated against human oral squamous carcinoma HSC-2, SCC-9, and A253 cells and human renal tubular epithelial HK-2 cells by Cell Counting Kit-8 (CCK-8) assay. The HSC-2, SCC-9, and A253 cell lines were purchased from Cobioer Biosciences (Nanjing, China), and HK-2 cell line was from KeyGen Biotech (Nanjing, China). The cells were placed into 96-well plates. After 24 h, cells were treated with 0.1% DMSO or different doses of test compounds for 72 h at 37°C in a CO_2_ incubator. Subsequently, 100 μl of CCK-8 test solution was added and incubated for 3 h at 37°C. The absorbance value of each well was measured at 450 nm.

#### Cell Cycle Analysis

HSC-2 cells were incubated in six-well plates and then treated with 0.1% DMSO or different concentrations of test compounds for 48 h. Subsequently, cells were fixed with 70% ethanol at 4°C overnight. After that, cells were washed with phosphate-buffered saline (PBS) and stained with 100 μl of RNase A at 37°C for 30 min and then 400 μl of propidium iodide (PI) at 4°C for 30 min. The cell cycle was measured by flow cytometry.

#### Cell Apoptosis Analysis

HSC-2 cells were seeded into six-well plates, grown overnight at 37°C in a 5% CO_2_ incubator, and then treated with 0.1% DMSO or different concentrations of test compounds for 48 h. Cells were washed twice with ice-cold PBS and then suspended with 500 μl of binding buffer. Subsequently, 5 μl of Annexin V-APC and 5 μl of 7-AAD were added, and the cells were incubated at room temperature for 15 min in the dark. The samples were analyzed by flow cytometry.

#### Mitochondrial Membrane Potential Assay

HSC-2 cells were seeded on six-well plates and grown overnight at 37°C in a 5% CO_2_ incubator. Then, cells were incubated with 0.1% DMSO or different concentrations of compounds at 37°C for 48 h. Subsequently, cells were trypsinized, washed with PBS, and resuspended in incubation buffer containing JC-1 at 37°C for 20 min in a 5% CO_2_ incubator. The dyeing cells were rinsed, suspended in incubation PBS, and then analyzed d by flow cytometry.

#### AO/EB Double Staining Assay

HSC-2 cells were seeded on six-well plates and grown overnight at 37°C in a 5% CO_2_ incubator. Then, cells were incubated with 0.1% DMSO or different concentrations of compounds at 37°C for 48 h. Subsequently, cells were trypsinized, collected, washed with PBS, and stained with 100 μl of AO/EB dye at room temperature for 15 min in the dark. The dyeing cells were rinsed, suspended in incubation PBS, and then observed under a fluorescence microscope.

#### Immunofluorescence Assay

HSC-2 cells were seeded on 24-well plates, grown overnight at 37°C in a 5% CO_2_ incubator, and then incubated with 0.1% DMSO or different concentrations of compounds at 37°C for 48 h. Cells were washed with PBS, fixed with 4% paraformaldehyde, and then further permeabilized with 0.5% Triton X-100 for 10 min. Subsequently, cells were blocked for 20 min by 50–100 μl of goat serum albumin at room temperature and then treated with α-tubulin primary antibody at 4°C for 2 h. Then, cells were treated with the Alexa Fluor 488-labeled goat anti-rabbit fluorescence secondary antibody at 37°C for 1 h in the dark, and the cell nuclei were labeled by DAPI at room temperature for 5 min. Finally, cells were visualized under a confocal microscope.

#### Wound Healing Assay

HSC-2 cells were seeded on six-well plates and grown to 80% at 37°C in a 5% CO_2_ incubator. Then, cells were scratched using the tip of the pipette, then washed with PBS to remove the non-adherent cells, and incubated with 0.1% DMSO or different concentrations of compounds at 37°C for 48 h. The images of cell migration were photographed with an inverted fluorescent microscope at 0 and 48 h.

#### Western Blot Analysis

After being cultured, HSC-2 cells were treated with 0.1% DMSO or different concentrations of compounds at 37°C for 48 h. Then, cells were collected, washed twice with PBS, and lysed with cold lysis buffer, followed by centrifugation. Then, the total proteins were collected, and protein concentration was quantified using Bradford assay. Equal amounts of proteins were electrophoresed by 10% sodium dodecyl sulfate–polyacrylamide gel electrophoresis (SDS-PAGE) and transferred onto nitrocellulose (NC) membrane. Membranes were blocked with 5% non-fat milk for 2 h, then washed, and incubated with primary antibodies overnight at 4°C. After being washed, the membranes were treated with secondary antibodies at room temperature for 2 h and then visualized using an ECL Western blotting kit.

### Molecular Modeling

The crystal structure of tubulin (PDB code 1SA0) was downloaded from the Protein Data Bank and selected as template. Molecular docking calculation was investigated using the DOCK 6.9 protocol in Yinfo Cloud Platform (http://cloud.yinfotek.com). The substrate **12b** was prepared with adding polar hydrogen atoms and partial charges, and further energy minimization in MMFF94 force field. And the protein tubulin was assigned polar hydrogen atoms and partial charges in Amber ff14SB force field and optimized by removing surplus structures and molecules. The DOCK 6.9 program was used to conduct flexible docking. The results were further analyzed using PyMOL.

### Statistical Analysis

All data were expressed as mean ± standard deviation (SD) and analyzed using SPSS 17.0 software. Differences between experimental groups and control groups were analyzed by Student's *t*-test. *P* < 0.05 was regarded as significant.

## Results and Discussion

### Chemistry

**Scheme 1:**
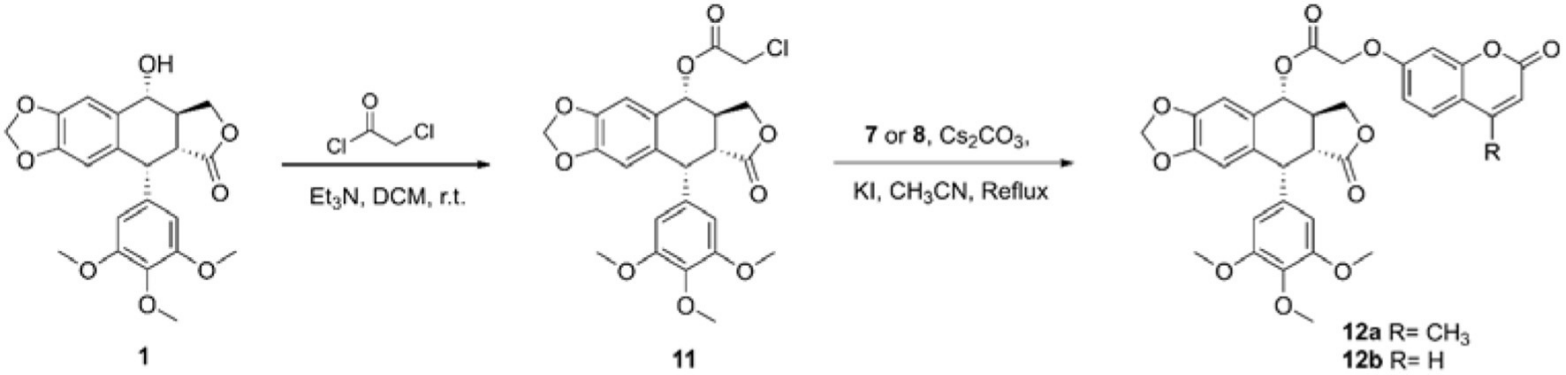
Synthesis of hybrids of podophyllotoxin and coumarin.

The synthetic route of hybrids of podophyllotoxin and coumarin is depicted in Scheme 1. The key intermediate **11** was obtained by the esterification reaction of podophyllotoxin (**1**) and chloroacetyl chloride. The target hybrids **12a–b** were synthesized via nucleophilic substitution reaction of coumarin (**7** or **8**) and intermediate **11** in the presence of cesium carbonate and catalytic amount potassium iodide. The conjugates were further characterized by ^1^H and ^13^C NMR spectrometry, and HRMS. The purity of final compound was determined by HPLC.

### Biological Evaluation

#### In vitro Anticancer Activity

The antiproliferative activities of target compounds (**12a–b**), lead compound podophyllotoxin (**1**), 4-methylumbelliferone (**7**), and 7-hydroxycoumarin (**8**) against human oral squamous carcinoma HSC-2, SCC-9, and A253 cells and human renal tubular epithelial HK-2 cells were screened using CCK-8 assay *in vitro*. 5-FU, etoposide, and cisplatin were used as positive compounds. The IC_50_ values were calculated and shown in Table 1. Firstly, two hybrids exhibited significant cytotoxicity against all three human oral cancer cell lines with IC_50_ values varying from 0.226 ± 0.021 to 0.349 ± 0.063 μM, which were more potent than positive compounds 5-FU, etoposide, and cisplatin. Moreover, compound **12b** containing the 7-hydroxycoumarin group possessed more potent cytotoxicity than **12a** against HSC-2, SCC-9, and A253 cells with IC_50_ values of 0.226 ± 0.021, 0.231 ± 0.05, and 0.25 ± 0.019 μM, respectively. As lead compounds, 4-methylumbelliferone (**7**) and 7-hydroxycoumarin (**8**) were observed to have no antiproliferative effect on all three oral cancer cell lines. And as expected, lead podophyllotoxin displayed excellent antiproliferative activity against not only all human oral cancer cell lines but also human normal cell line. Although the IC_50_ values of podophyllotoxin against all human oral cancer cell lines were stronger than **12b**, **12b** showed less inhibition effect (IC_50 =_ 0.626 ± 0.043 μM) on the viability of HK-2 cells than podophyllotoxin (IC_50 =_ 0.04 ± 0.006 μM), which indicated that **12b** had good selectivity between tumor and normal cells, and it exhibited much less toxicity on human normal cells. Moreover, hybrid **12b** in the present study showed more anticancer potential than the conjugates of 4′-demethylepipodophyllotoxin and coumarin (Hao et al., 2019).

**Table 1:** Antiproliferative activity *in vitro*.

**Compd**.	**IC**_****50****_ **(μM)**^****a****^			
	**HSC-2**	**SCC-9**	**A253**	**HK-2**
12a	0.281 ± 0.024	0.279 ± 0.041	0.349 ± 0.063	0.814 ± 0.173
12b	0.226 ± 0.021	0.231 ± 0.05	0.25 ± 0.019	0.626 ± 0.043
7	>100	>100	>100	NT
8	>100	>100	>100	NT
1	0.011 ± 0.003	0.009 ± 0.001	0.01 ± 0.001	0.04 ± 0.006
5-FU	7.117 ± 0.87	5.347 ± 0.784	5.52 ± 0.199	8.769 ± 1.036
Etoposide	1.246 ± 0.467	1.15 ± 0.308	0.95 ± 0.387	3.039 ± 1.371
Cisplatin	2.03 ± 0.308	2.353 ± 0.384	1.617 ± 0.157	5.36 ± 0.33

*NT, not tested; CCK-8, Cell Counting Kit-8*.

a *Cells were treated with compounds by CCK-8 assay for 72 h. IC_50_ values are indicated as mean IC_50_ ± SD (μM)*.

#### Cell Cycle Analysis

**Figure 3:**
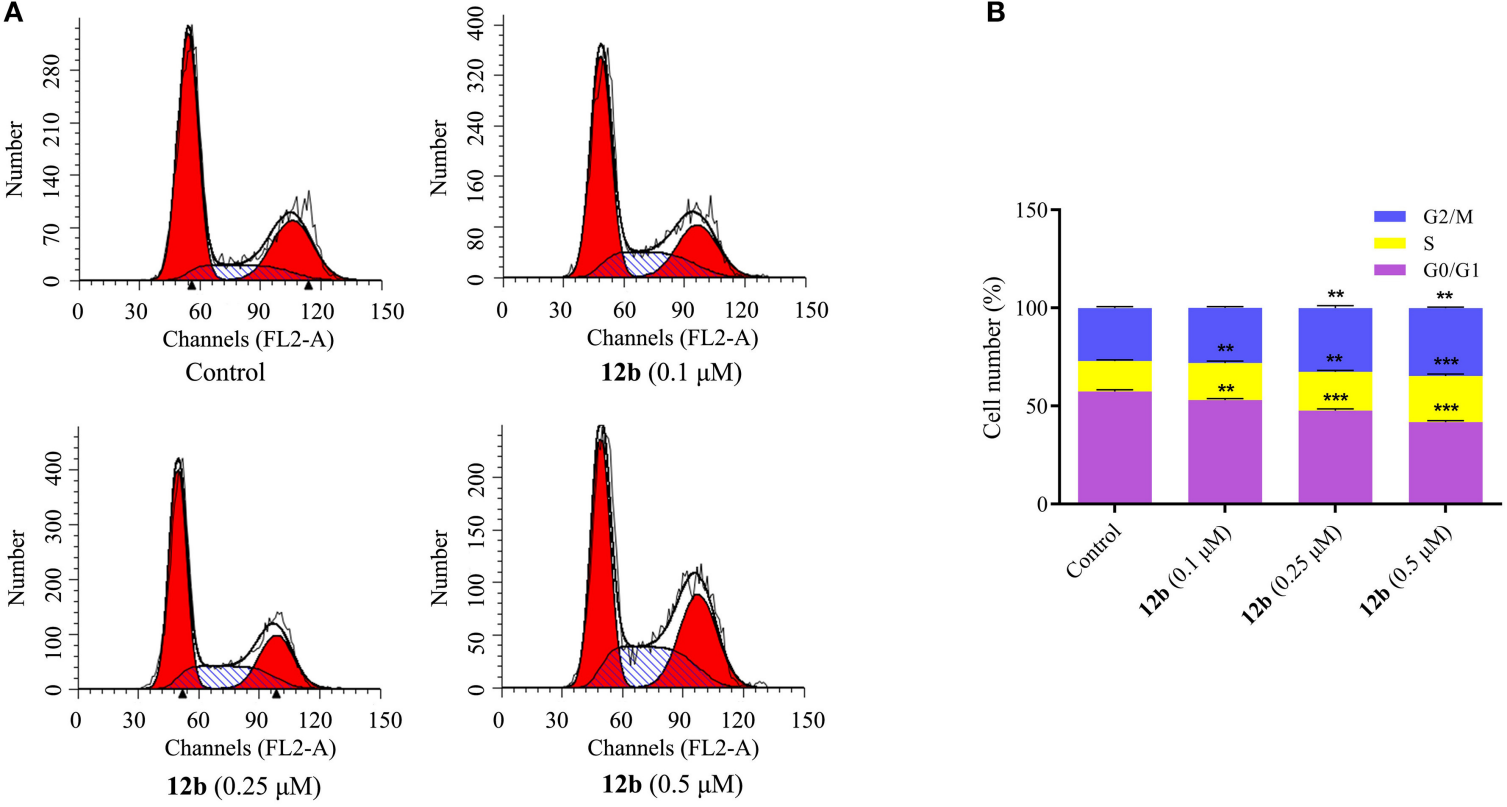
Effects of **12b** on the cell cycle. HSC-2 cells were incubated with vehicle and **12b** (0.1, 0.25, and 0.5 μM) for 48 h. Then, the cell cycle distribution was further analyzed by flow cytometry after labeling with propidium iodide (PI). Data are expressed as mean ± SD. **(A)** Flow cytometry analysis; **(B)** quantitative analysis. **P < 0.01 and ***P < 0.001 vs. the control group.

To detect whether cytotoxicity potency of compound **12b** resulted from cell cycle progression, the effects of **12b** on the cell cycle of HSC-2 cells were analyzed by flow cytometry after labeling with PI. HSC-2 cells were incubated with vehicle and **12b** (0.1, 0.25, and 0.5 μM) for 48 h. As seen in Figure 3, compared with the control group, treatment of HSC-2 cells with 0.1 μM of **12b** showed a significant S accumulation, while higher concentrations (0.25 and 0.5 μM) of **12b** caused cell cycle arrest at both S and G2 phases in a dose-dependent manner. In short, the cell cycle arrest of **12b** could be responsible for the cytotoxicity in cancer cells.

#### Apoptosis Analysis

**Figure 4:**
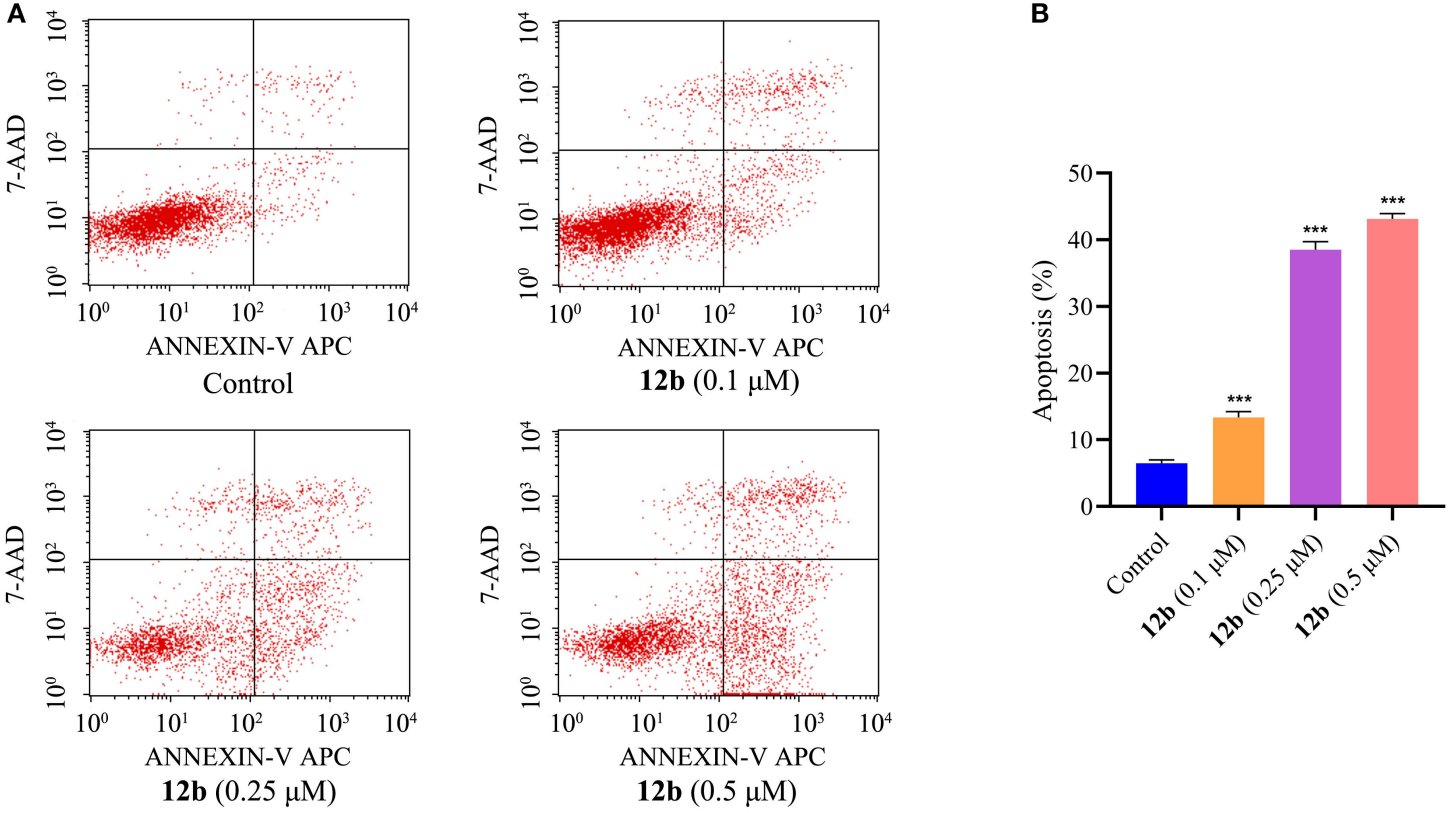
Effects of **12b** on the induction of apoptosis in HSC-2 cells. Cells were incubated with vehicle and **12b** (0.1, 0.25, and 0.5 μM) for 48 h. Then, the percentages of apoptotic HSC-2 cells were analyzed by flow cytometry after labeling with Annexin V-FITC/7-AAD. Data are expressed as mean ± SD. **(A)** Flow cytometry analysis; **(B)** quantitative analysis. ***P < 0.001 vs. the control group.

Further efforts were conducted to conform whether the cytotoxicity of compound **12b** in the oral cancer cells was related to the induction of apoptosis. The effects of **12b** on the induction of apoptosis in HSC-2 cells were analyzed by flow cytometry after labeling with Annexin V-FITC/7-AAD. Cells were incubated with vehicle and **12b** (0.1, 0.25, and 0.5 μM) for 48 h. As shown in Figure 4, compared with the control group, **12b** obviously induced apoptosis in HSC-2 cells in a dose-dependent manner.

#### Mitochondrial Membrane Potential Analysis

**Figure 5:**
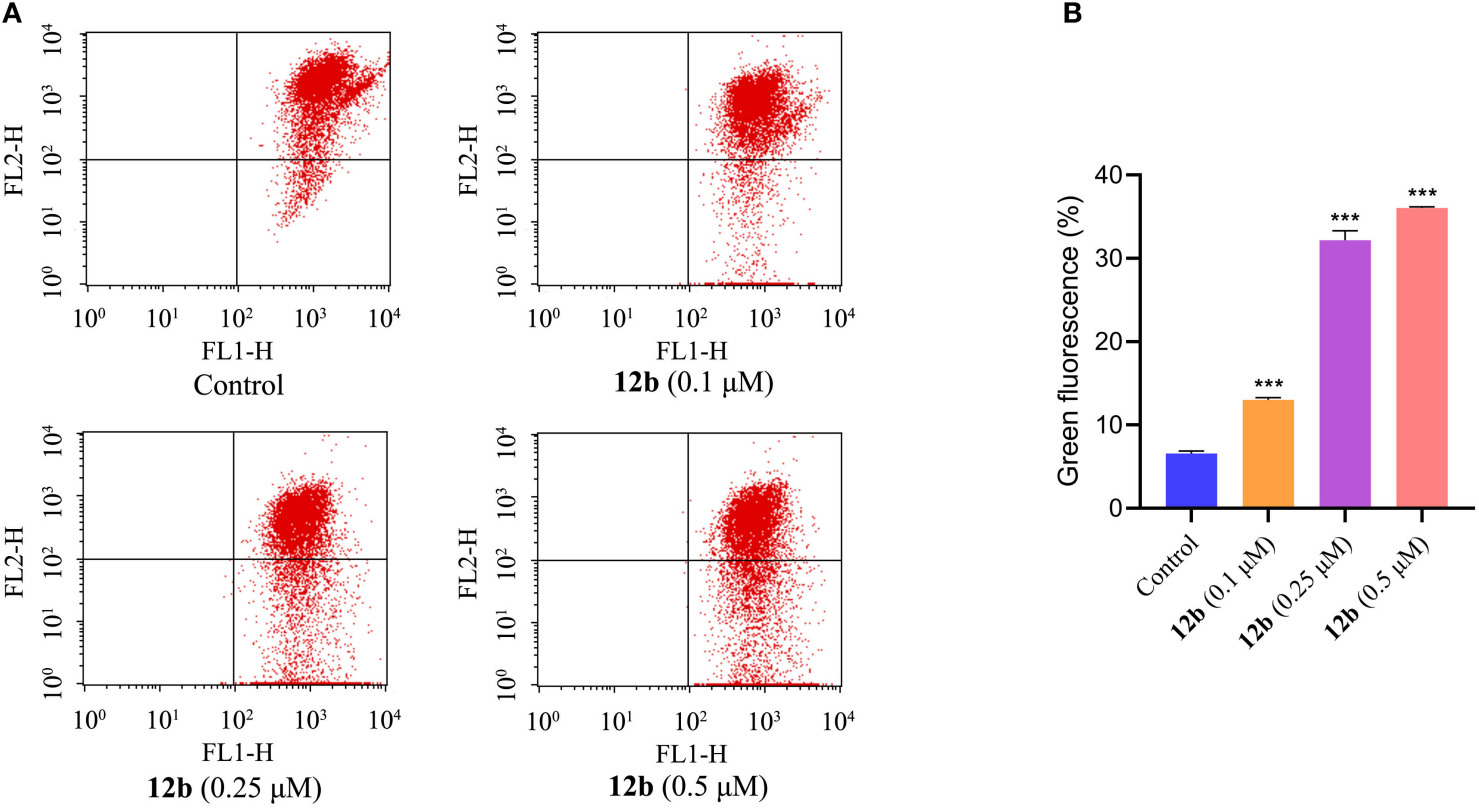
Effects of **12b** on the mitochondrial membrane potential in HSC-2 cells. Cells were incubated with vehicle and **12b** (0.1, 0.25, and 0.5 μM) for 48 h. Then, cells were dyed with JC-1 and further analyzed by flow cytometry. Data are expressed as mean ± SD. **(A)** Flow cytometry analysis; **(B)** quantitative analysis. ***P < 0.001 vs. the control group.

In order to further confirm the effects of **12b** on the mitochondrial membrane potential (MMP), which is an important marker of apoptosis (Tsujimoto and Shimizu, 2007), fluorescent probe JC-1 was used to detect the MMP. HSC-2 cells were incubated with vehicle and **12b** (0.1, 0.25, and 0.5 μM) for 48 h, and then cells were dyed with JC-1 and further analyzed by flow cytometry. As shown in Figure 5, compared with the control group, MMP was significantly decreased in HSC-2 cells treated with **12b** in a dose-dependent manner.

#### AO/EB Double Staining

**Figure 6:**
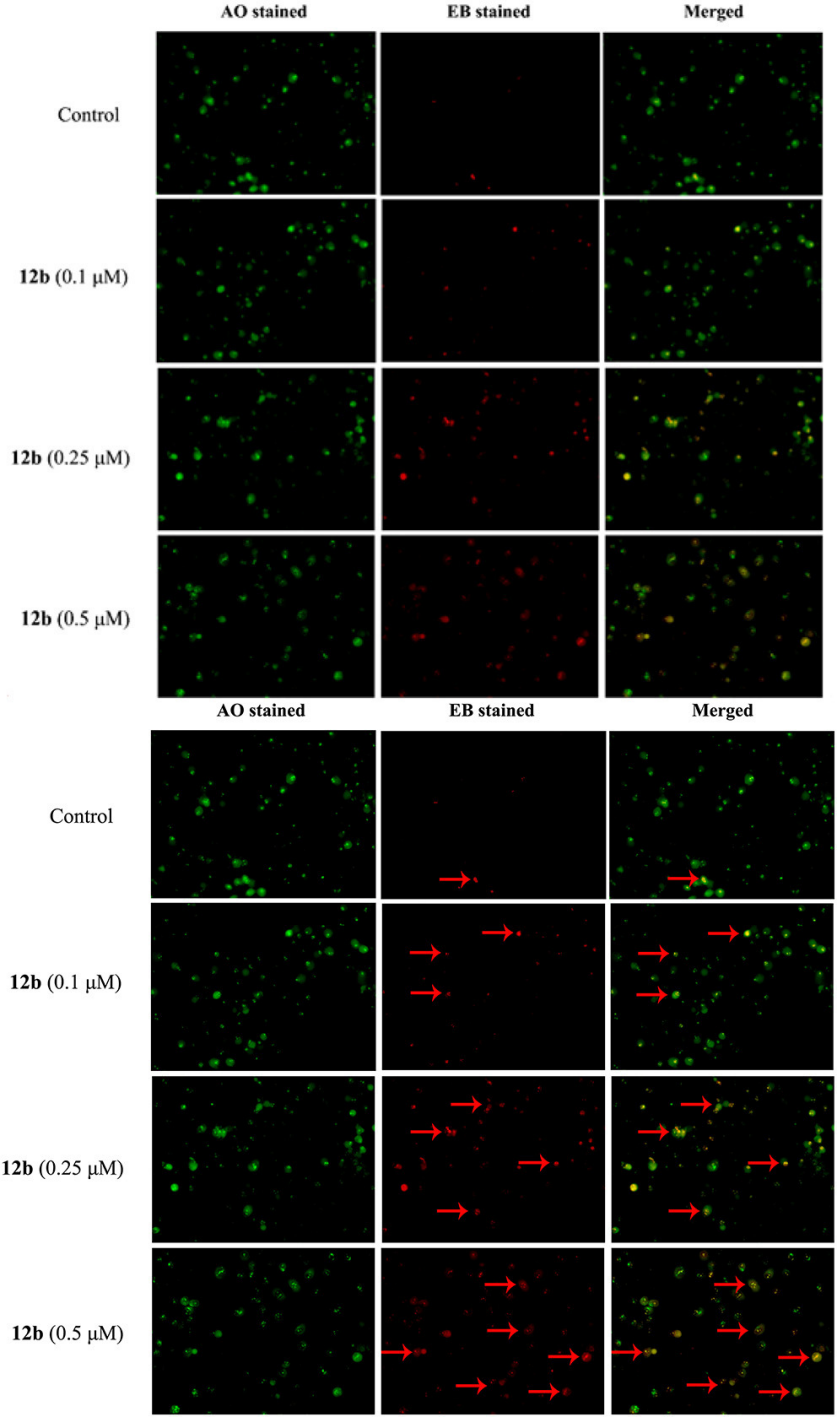
AO/EB staining assay analysis. HSC-2 cells were incubated with vehicle and **12b** (0.1, 0.25, and 0.5 μM) for 48 h and then stained with AO/EB. The live cells (green) and apoptotic cells (red) were tested by fluorescence microscopy. Arrows indicate the apoptotic cells with morphological changes.

As compound **12b** induced HSC-2 cells death via the apoptotic pathway, we further evaluated the morphological change using AO/EB staining assay. HSC-2 cells were treated with vehicle and **12b** (0.1, 0.25, and 0.5 μM) for 48 h, then stained with AO/EB, and tested by fluorescence microscopy. As depicted in Figure 6, few cells underwent apoptosis in the control group; however, after treatment with **12b**, the cells were stained to light-orange fluorescence and showed chromatin condensation and shrinkage, indicating that **12b** could induce apoptosis in HSC-2 cells.

#### Immunofluorescent Analysis

**Figure 7:**
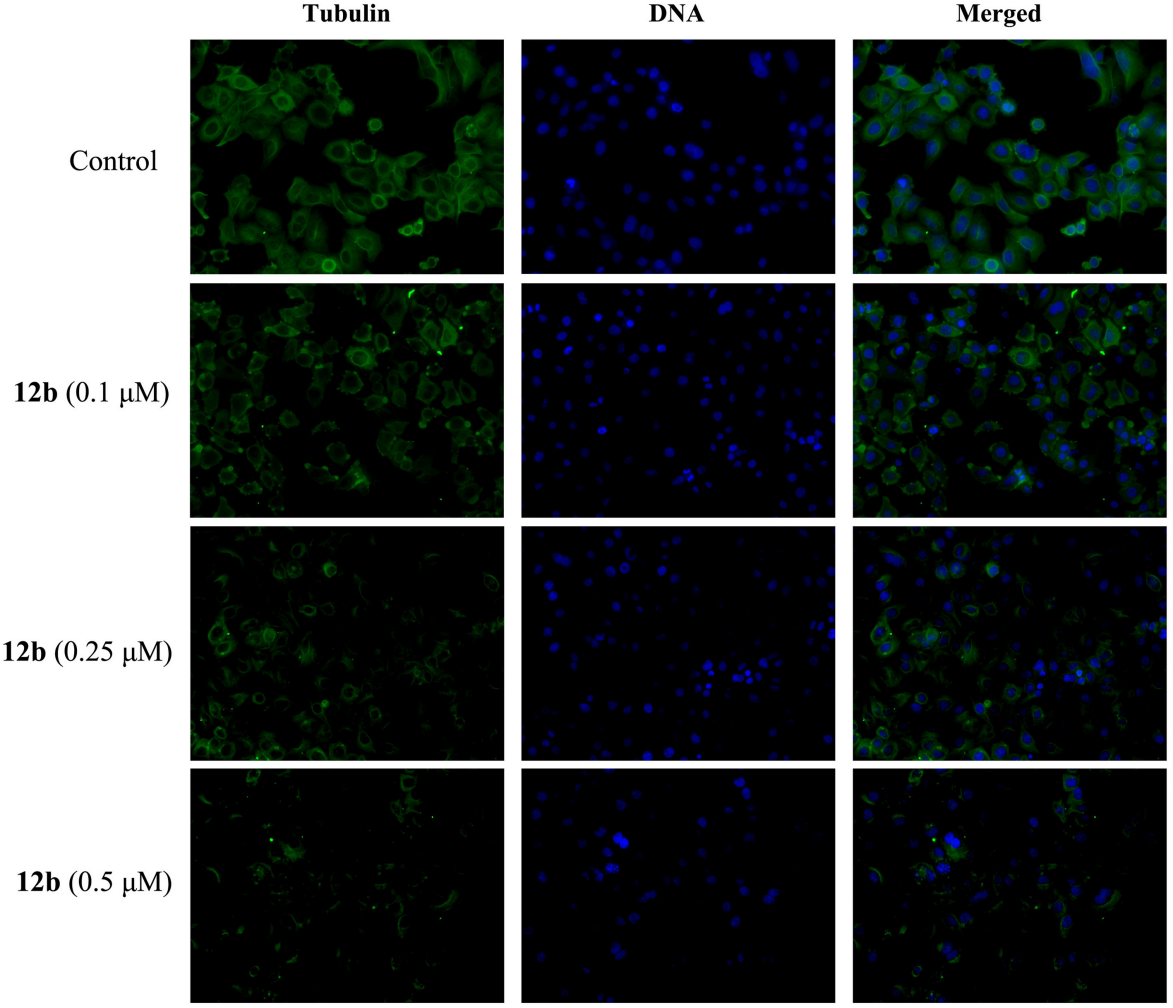
Effects of **12b** on the organizations of microtubule network in HSC-2 cells by immunofluorescence assay analysis. Cells were incubated with vehicle and **12b** (0.1, 0.25, and 0.5 μM) for 48 h. The microtubules were dyed with anti-tubulin antibody (green), and cell nuclei were stained with DAPI (blue). Images were captured using LSM 710 laser confocal microscope.

To study whether the hybrid **12b** was able to disrupt the organizations of microtubule network in HSC-2 cells, we investigated the effect of **12b** on the microtubule network by immunofluorescence assay analysis. Cells were incubated with vehicle and **12b** (0.1, 0.25, and 0.5 μM) for 48 h. The microtubules were dyed with anti-tubulin antibody, and cell nuclei were stained with DAPI. As shown in Figure 7, the microtubule network in the control cells exhibited normal organization with slim and fibrous microtubules (green) around the cell nucleus (blue). However, in the **12b**-treated cells, intracellular microtubules became notably stabilized and disordered in a dose-dependent manner, indicating the disruption of microtubule network organizations. Taken together, these data indicated that **12b** could induce the depolymerization of the microtubule network, which may lead to cell cycle arrest and apoptosis.

### Molecular Modeling

**Figure 8:**
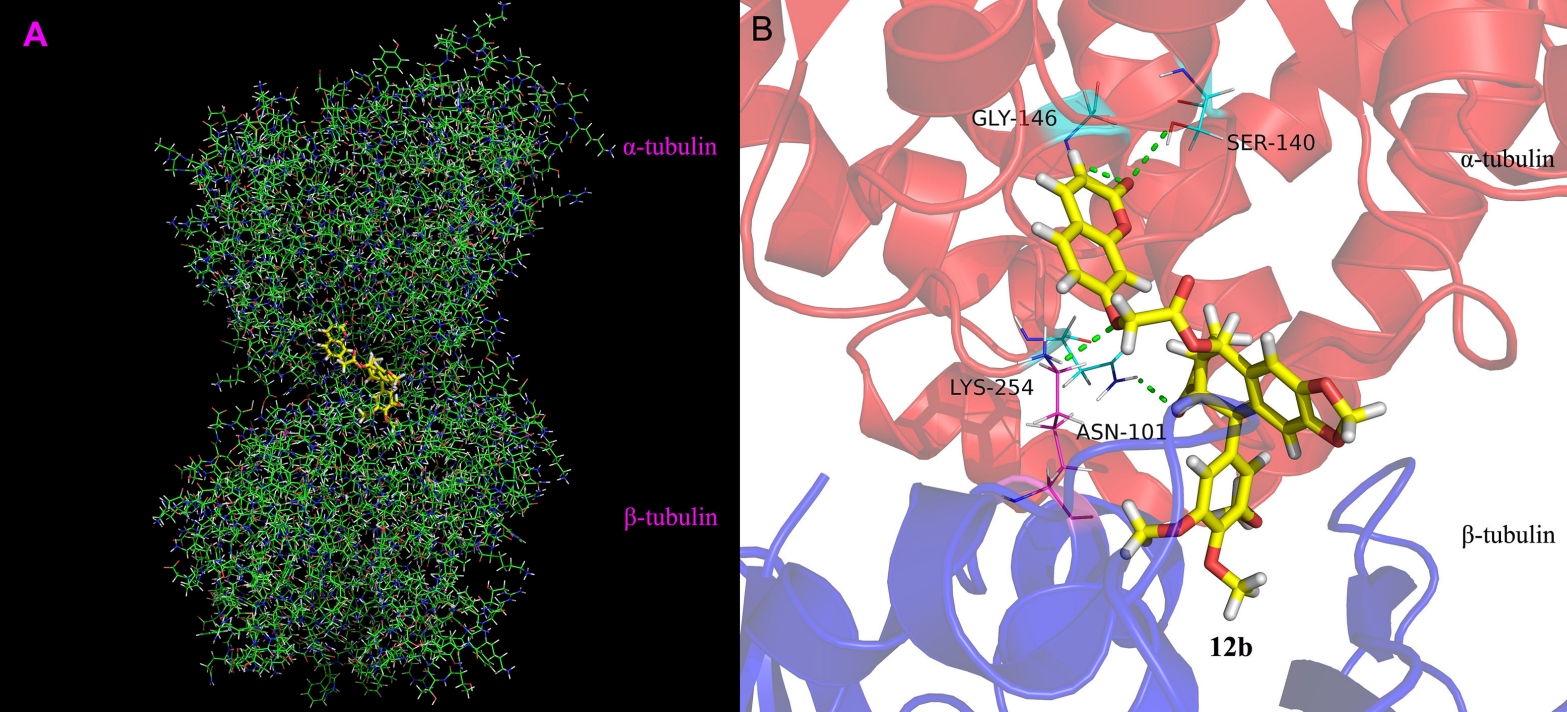
Binding mode of **12b** in the colchicine site of tubulin. **(A)** Docking model of **12b** (yellow color stick) and **(B)** ligand–protein interactions. The green dashed lines represent the hydrogen bonds.

To explore the binding mode of **12b** and tubulin, molecular docking calculation was investigated of **12b** into the colchicine binding pocket of tubulin (PDB code: 1SA0). As shown in Figure 8, the results indicated that 3,4,5-trimethoxyphenyl group (E ring) of **12b** is located deeply into the colchicine binding site in the β-subunit of tubulin. The oxygen of the carbonyl in lactone ring could form one hydrogen bond with –NH in ASN-101, and the hydrogen bond distance was 2.1 Å. Moreover, the coumarin group was located in the hydrophobic pocket of α-subunit of tubulin. The 7-O of coumarin group formed one hydrogen bond with –NH in LYS-254 (*d* = 2.5 Å). In addition, the oxygen of the carboxide in the coumarin group had two hydrogen bonds with the –NH in GLY-146 and –OH in SER-140 with distances of 2.4 and 2.9 Å, respectively. Overall, molecular docking study indicated that **12b** could bind into the colchicine site of tubulin.

#### Transwell Invasion Assay

**Figure 9:**
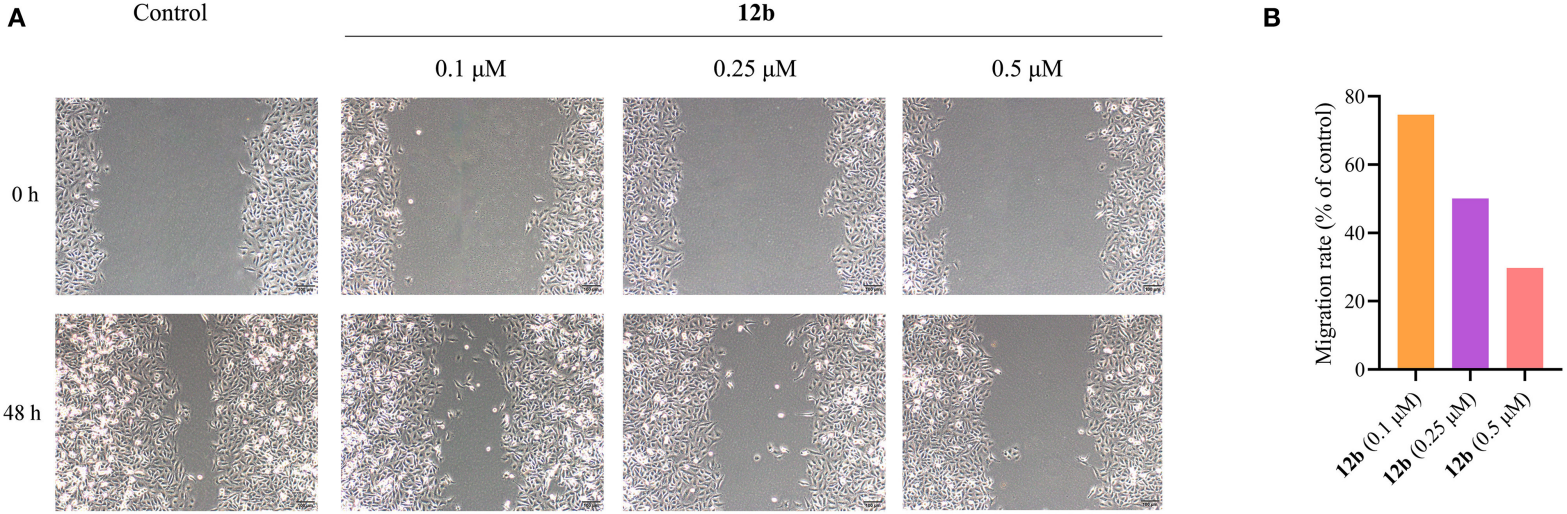
Wound healing migration assay of HSC-2 cells. **(A)** Cells were incubated with vehicle and **12b** (0.1, 0.25, and 0.5 μM) for 48 h. The images were photographed at 0 and 48 h. **(B)** Relative inhibition of cell migration. The percentage of inhibition was calculated relative to control group.

Then, we further investigated the effect of **12b** on the migration of HSC-2 cells using wound healing assay. Cells were incubated with vehicle and **12b** (0.1, 0.25, and 0.5 μM) for 48 h. The images were photographed at 0 and 48 h. And the migration rates of test compounds were quantified and calculated relative to untreated cells. In wound healing assay (Figure 9), compared with the control group, **12b** inhibited HSC-2 cell migration in a dose-dependent manner. These results indicated that **12b** had the ability to inhibit oral cancer cell migration.

#### Western Blotting

**Figure 10:**
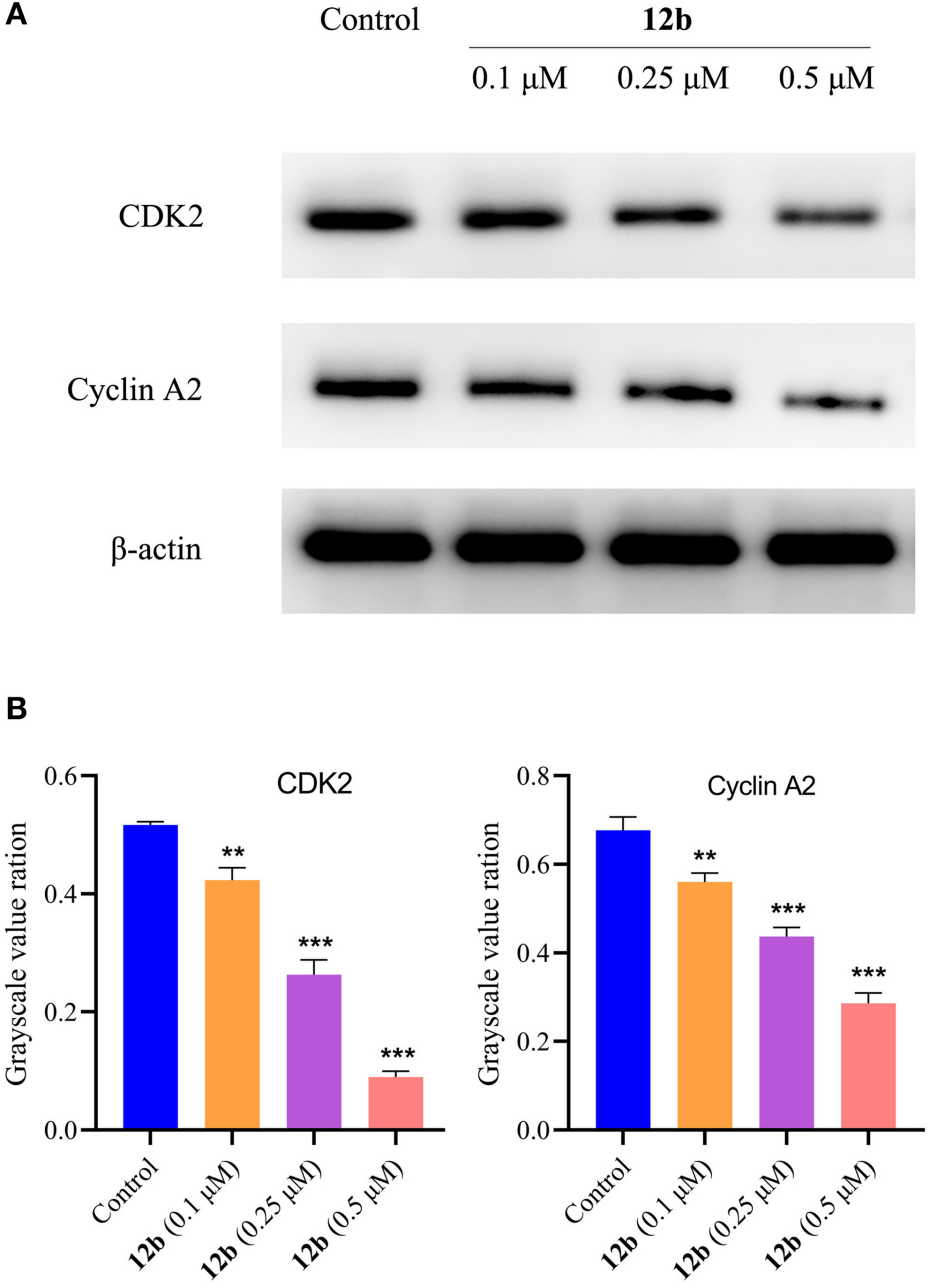
Effects of **12b** on the cycle-related proteins. HSC-2 cells were incubated with vehicle and **12b** (0.1, 0.25, and 0.5 μM) for 48 h. Western blot was used to detect the relative levels of CDK2 and cyclin A2, and β-actin was used as internal standard. Data are expressed as mean ± SD. **(A)** Western blot analysis; **(B)** quantitative analysis. **P < 0.01 and ***P < 0.001 vs. the control group.

Next, we explored the molecular mechanism behind **12b**-induced cell cycle arrest in HSC-2 cells. Cells were incubated with vehicle and **12b** (0.1, 0.25, and 0.5 μM) for 48 h. Western blot was used to detect the relative levels of cycle-related proteins CDK2 and cyclin A2, which were essential for driving S and G2 cell-cycle phases (Hochegger et al., 2008), and β-actin was used as internal standard. Compared with the control group, the CDK2 and cyclin A2 in **12b**-treated HSC-2 cells were suppressed in a concentration-dependent manner (Figure 10). Interestingly, a low concentration (0.1 μM) of **12b** slightly inhibited the expression of CDK2 and cyclin A2, while higher concentrations (0.25 and 0.5 μM) of **12b** significantly downregulated the levels of CDK2 and cyclin A2 in HSC-2 cells, which maybe cause the above results that a low concentration (0.1 μM) of **12b** showed a significant S accumulation and higher concentrations (0.25 and 0.5 μM) of **12b** caused cell cycle arrest at both S and G2 phases.

**Figure 11:**
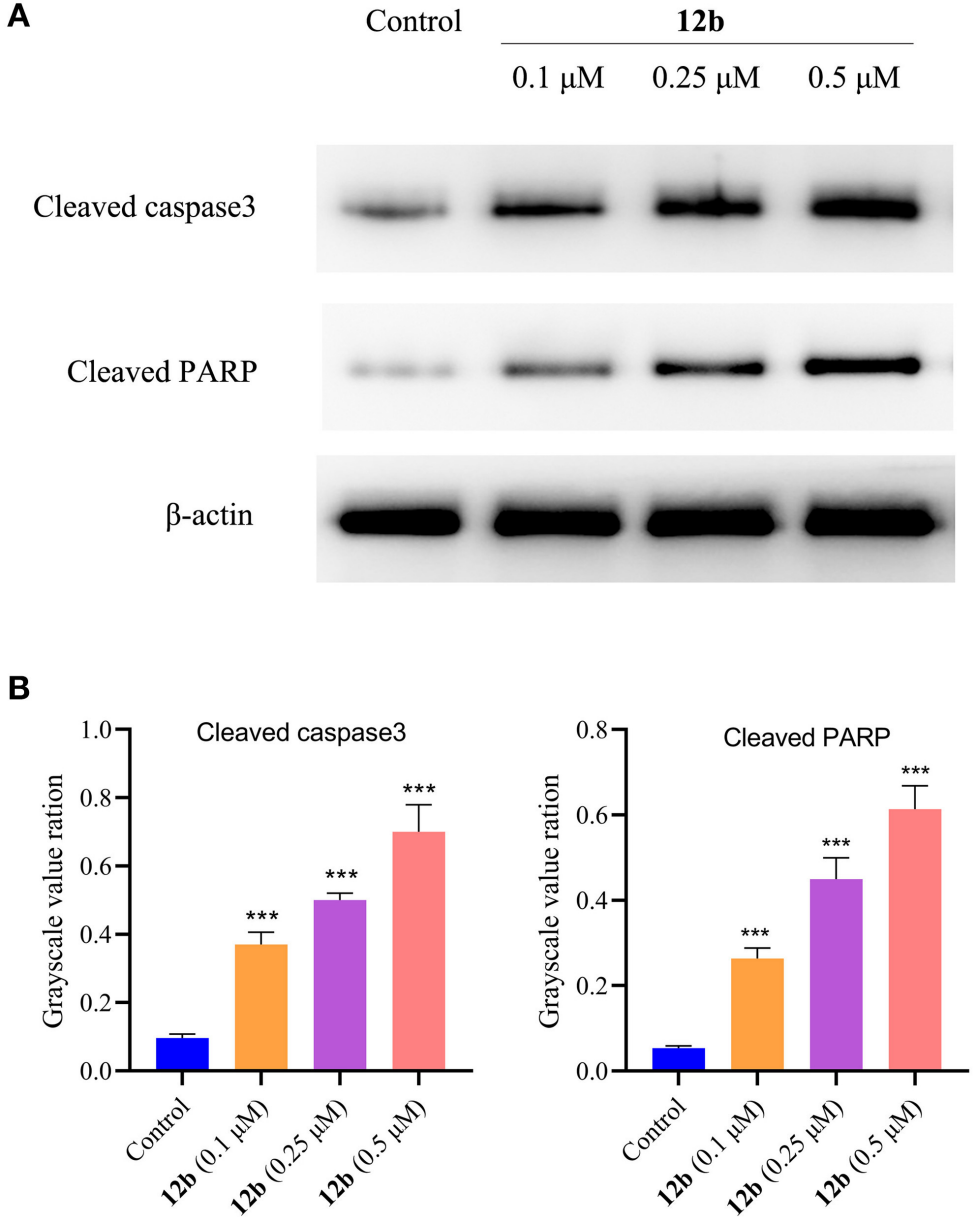
Effects of **12b** on the apoptosis-related proteins. HSC-2 cells were incubated with vehicle and **12b** (0.1, 0.25, and 0.5 μM) for 48 h. Western blot was used to detect the relative levels of cleaved caspase3 and cleaved PARP, and β-actin was used as internal standard. Data are expressed as mean ± SD. **(A)** Western blot analysis; **(B)** quantitative analysis. ***P < 0.001 vs. the control group.

In order to characterize the molecular mechanisms of **12b**-induced apoptosis, we explored the expression levels of cleaved caspase3 and cleaved PARP, two critical apoptosis-related proteins. HSC-2 cells were incubated with vehicle and **12b** (0.1, 0.25, and 0.5 μM) for 48 h. Western blot was used to detect the relative levels of cleaved caspase3 and cleaved PARP. As shown in Figure 11, we observed that **12b** upregulated the relative levels of cleaved caspase3 and cleaved PARP in a concentration-dependent manner.

**Figure 12:**
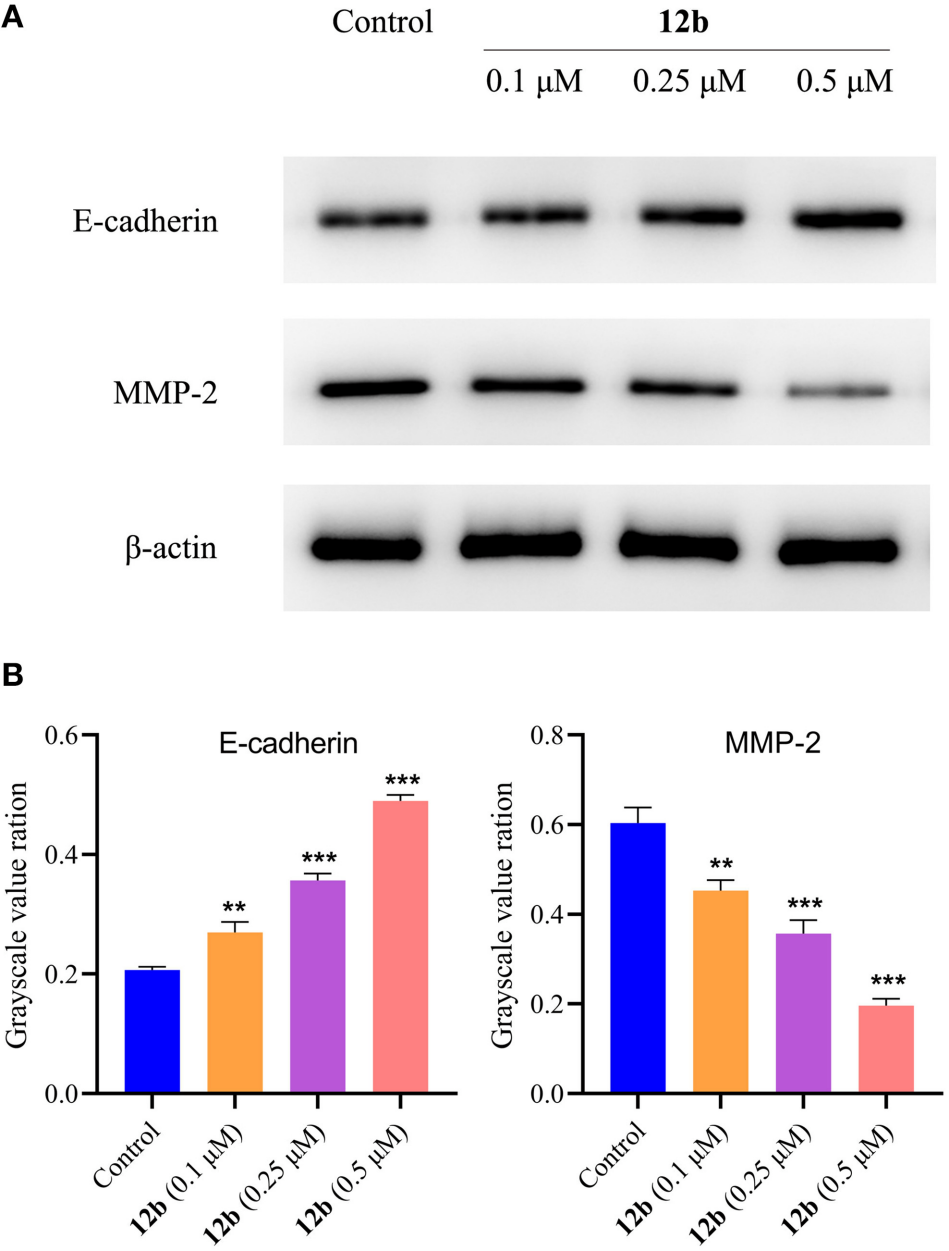
Effects of **12b** on the migration-related proteins. HSC-2 cells were incubated with vehicle and **12b** (0.1, 0.25, and 0.5 μM) for 48 h. Western blot was used to detect the relative levels of E-cadherin and MMP-2, and β-actin was used as internal standard. Data are expressed as mean ± SD. **(A)** Western blot analysis; **(B)** quantitative analysis. **P < 0.01 and ***P < 0.001 vs. the control group.

As mentioned above, **12b** inhibited HSC-2 cell migration in wound healing assay. Thus, we evaluated the effects of **12b** on the migration-related proteins in HSC-2 cells. Cells were incubated with vehicle and **12b** (0.1, 0.25, and 0.5 μM) for 48 h. Western blot was used to detect the relative levels of E-cadherin and MMP-2, which were associated with tumor proliferation and metastasis (Grelewski and Bar, 2013; Hu et al., 2016). As shown in Figure 12, it was observed that **12b** obviously enhanced the expression of E-cadherin and reduced the expression of MMP-2 compared with the control group. These data showed that the antimigratory effects of **12b** on HSC-2 cells were mediated by regulating the expression of E-cadherin and MMP-2.

**Figure 13:**
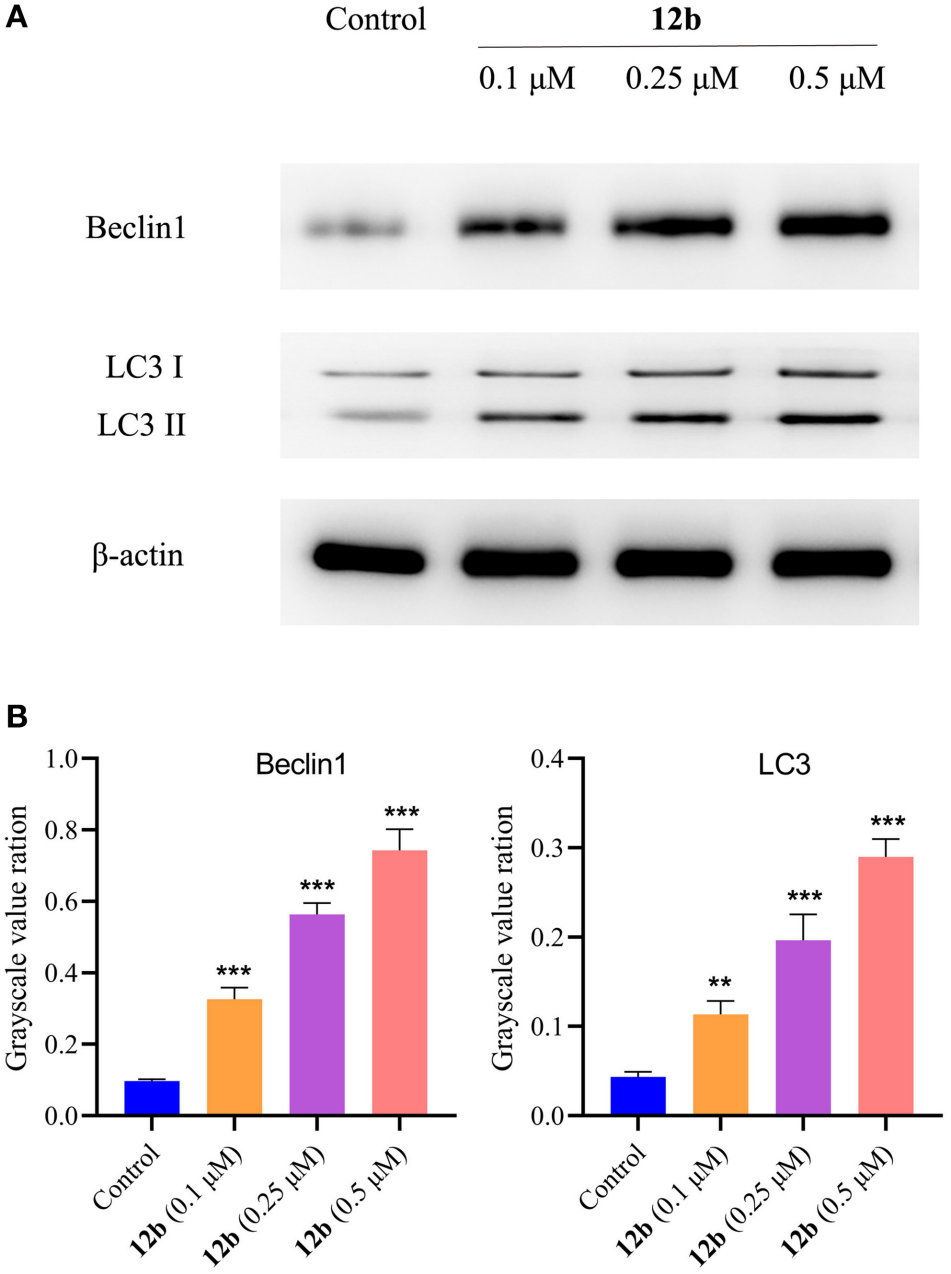
Effects of **12b** on the autophagy-related proteins. HSC-2 cells were incubated with vehicle and **12b** (0.1, 0.25, and 0.5 μM) for 48 h. Western blot was used to detect the relative levels of Beclin1 and LC3, and β-actin was used as internal standard. Data are expressed as mean ± SD. **(A)** Western blot analysis; **(B)** quantitative analysis. **P < 0.01 and ***P < 0.001 vs. the control group.

Considering autophagy is another crucial programmed cell death (Levy and Thorburn, 2020), we next evaluated whether autophagy was involved in **12b**-reduced HSC-2 cell death. Cells were incubated with vehicle and **12b** (0.1, 0.25, and 0.5 μM) for 48 h. Western blot was used to detect the relative levels of Beclin1 and LC3. It was found that **12b** significantly induced autophagy by increasing the aggregation of Beclin1 and LC3 (Figure 13). Moreover, we observed that **12b** upregulated the level of cleavage of LC3 (LC3-II). These results indicated that **12b** induced autophagy in HSC-2 cells.

**Figure 14:**
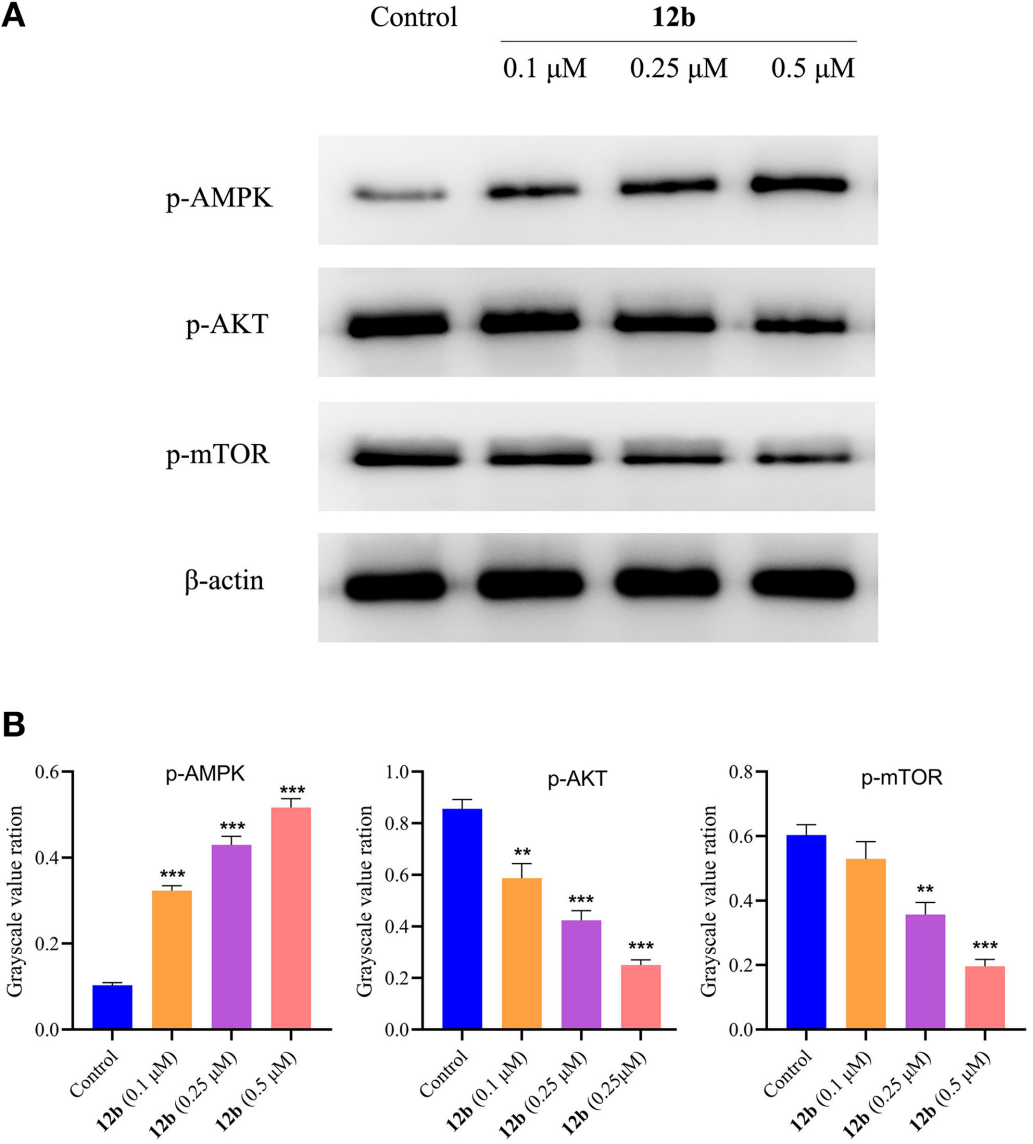
Effects of **12b** on the AMPK, AKT, and mTOR signaling. HSC-2 cells were incubated with vehicle and **12b** (0.1, 0.25, and 0.5 μM) for 48 h. Western blot was used to detect the relative levels of p-AMPK, p-AKT, and p-mTOR; and β-actin was used as internal standard. Data are expressed as mean ± SD. **(A)** Western blot analysis; **(B)** quantitative analysis. **P < 0.01 and ***P < 0.001 vs. the control group.

Many signaling pathways, such as AMPK and AKT/mTOR, play important roles in the regulation of autophagy and maintenance of cellular homeostasis and survival. AMPK acted as a positive regulator of autophagy (Mihaylova and Shaw, 2011), whereas AKT/mTOR pathway was reported to contribute to inhibiting autophagy (Xu et al., 2020). According to the above results, we next detected the effects of **12b** on the autophagy-related pathways in HSC-2 cells. Cells were incubated with vehicle and **12b** (0.1, 0.25, and 0.5 μM) for 48 h. Western blot was used to detect the relative levels of p-AMPK, p-AKT, and p-mTOR. As shown in Figure 14, we found that p-AMPK expression was upregulated in HSC-2 cells following incubation with **12b** in a dose-dependent manner. Further investigation of the AKT/mTOR signaling pathway indicated that **12b** downregulated phosphorylation of AKT, leading to downstream inhibition of mTOR phosphorylation in HSC-2 cells.

## Conclusions

Overall, in present study, two novel hybrids of podophyllotoxin and coumarins were designed and synthesized using structure-based and molecular hybridization strategies. According to the results of biological evaluation, it was observed that the hybrid of podophyllotoxin and 7-hydroxycoumarin (**12b**) showed better antiproliferative activities in three human oral squamous carcinoma cell lines than clinical drugs and exhibited less toxicity than podophyllotoxin in human normal cells. In addition, further molecular mechanism research indicated that **12b** was able to inhibit the growth of HSC-2 cells by inducing cell cycle arrest and apoptosis, as well as disturbing the MMP. Meanwhile, **12b** could inhibit cell migration and disorder the cellular microtubule network via binding to the tubulin, which was proved by molecular docking study. Western blot results demonstrated that **12b** inhibited the migration of HSC-2 cells and induced cell autophagy, which was associated with the regulation of AMPK and AKT/mTOR signaling pathways. This study expanded the structural diversity of podophyllotoxin analogs and was beneficial to the development of novel drug for the therapy of OSCC. Nevertheless, our data still need further evaluation to investigate the acute toxicity and stabilization of **12b**, as well as its anticancer efficacy in animal model.

## Data Availability Statement

The original contributions generated for the study are included in the article/Supplementary Material, further inquiries can be directed to the corresponding author/s.

## Author Contributions

GB, DZ, and XR performed the *in vitro* biological studies. GB wrote the whole manuscript. LZ was responsible for design, synthesis, and purification of the target compounds, as well as the docking study. D-GZ corrected the language and revised the manuscript. All of the authors approved the submitted version.

## Conflict of Interest

The authors declare that the research was conducted in the absence of any commercial or financial relationships that could be construed as a potential conflict of interest.
