# Supplementary material for: Novel Hybrids of Podophyllotoxin and Coumarin Inhibit the Growth and Migration of Human Oral Squamous Carcinoma Cells
Source: Front Chem. 2021 Jan 15;8:626075. doi: 10.3389/fchem.2020.626075 (PMC7843452; doi:10.3389/fchem.2020.626075)

Supplementary Material

Novel Hybrids of Podophyllotoxin and Coumarin Inhibit the Growth and Migration of Human Oral Squamous Carcinoma Cells

Guohui Bai^1^, Dan Zhao^1^, Xin Ran^1^, Lei Zhang^2*^ and Degang Zhao^1,3*^

^1^Institute of Agro-Bioengineering and College of Life Sciences, The Key Laboratory of Plant Resources Conservation and Germplasm Innovation in Mountainous Region (Ministry of Education), Guizhou University, Guiyang, China

^2^Key Laboratory of Biocatalysis & Chiral Drug Synthesis of Guizhou Province and School of Pharmacy, Zunyi Medical University, Zunyi, China

^3^Institute of Guizhou Distinctive Plant Resources Conservation, Guizhou Academy of Agricultural Science, Guiyang, China

*** Correspondence:**Lei Zhang
[lzhang@zmu.edu.cn](mailto:lzhang@zmu.edu.cn)

Degang Zhao
[dgzhao@gzu.edu.cn](mailto:dgzhao@gzu.edu.cn)

**Table of content**

1. ^1^H -NMR and ^13^C- NMR spectraof compound **11**
2. ^1^H -NMR, ^13^C- NMR spectra and HR-MS of compounds **12a-b**
3. Table S1: Purity data of final compounds (HPLC)


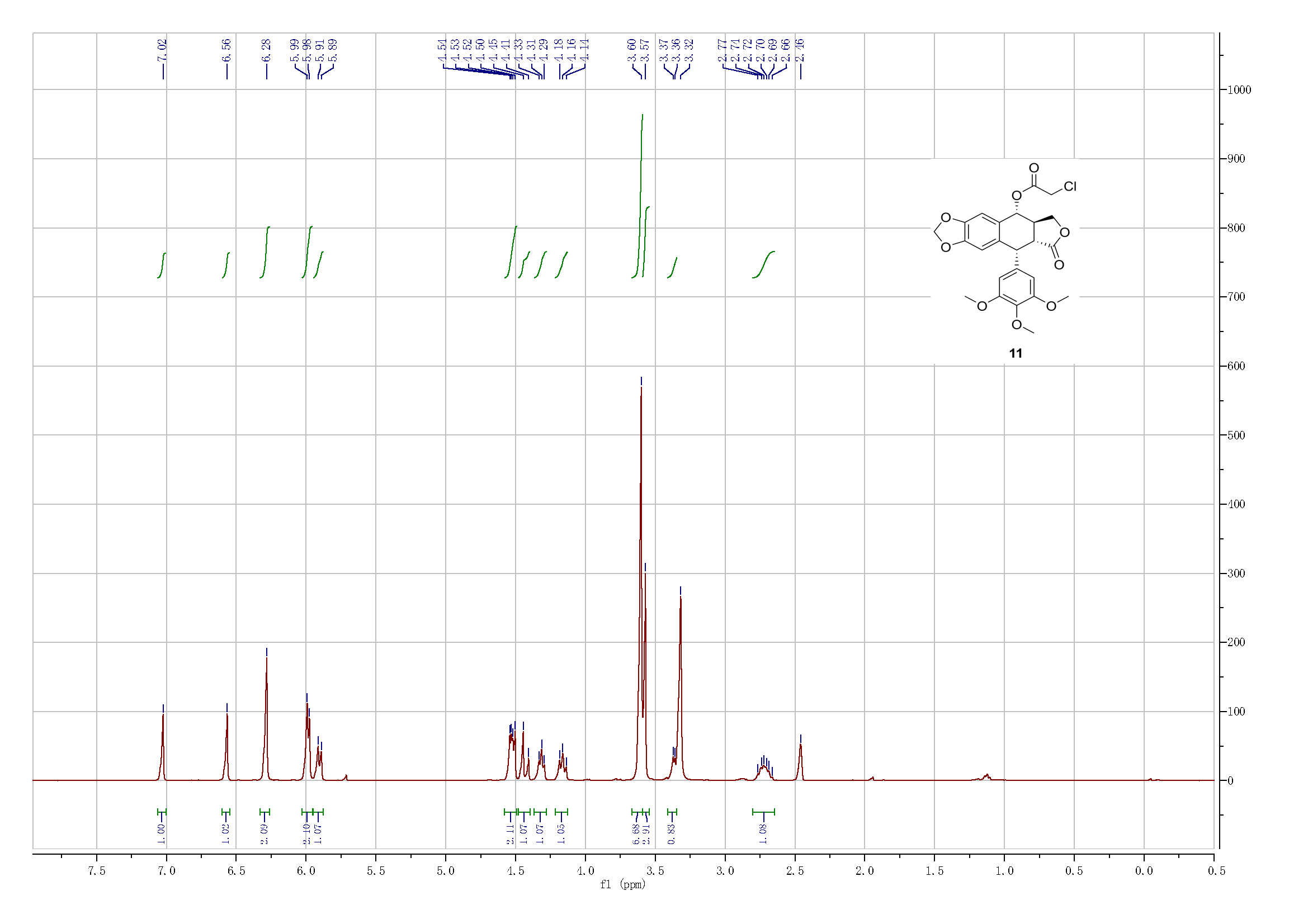


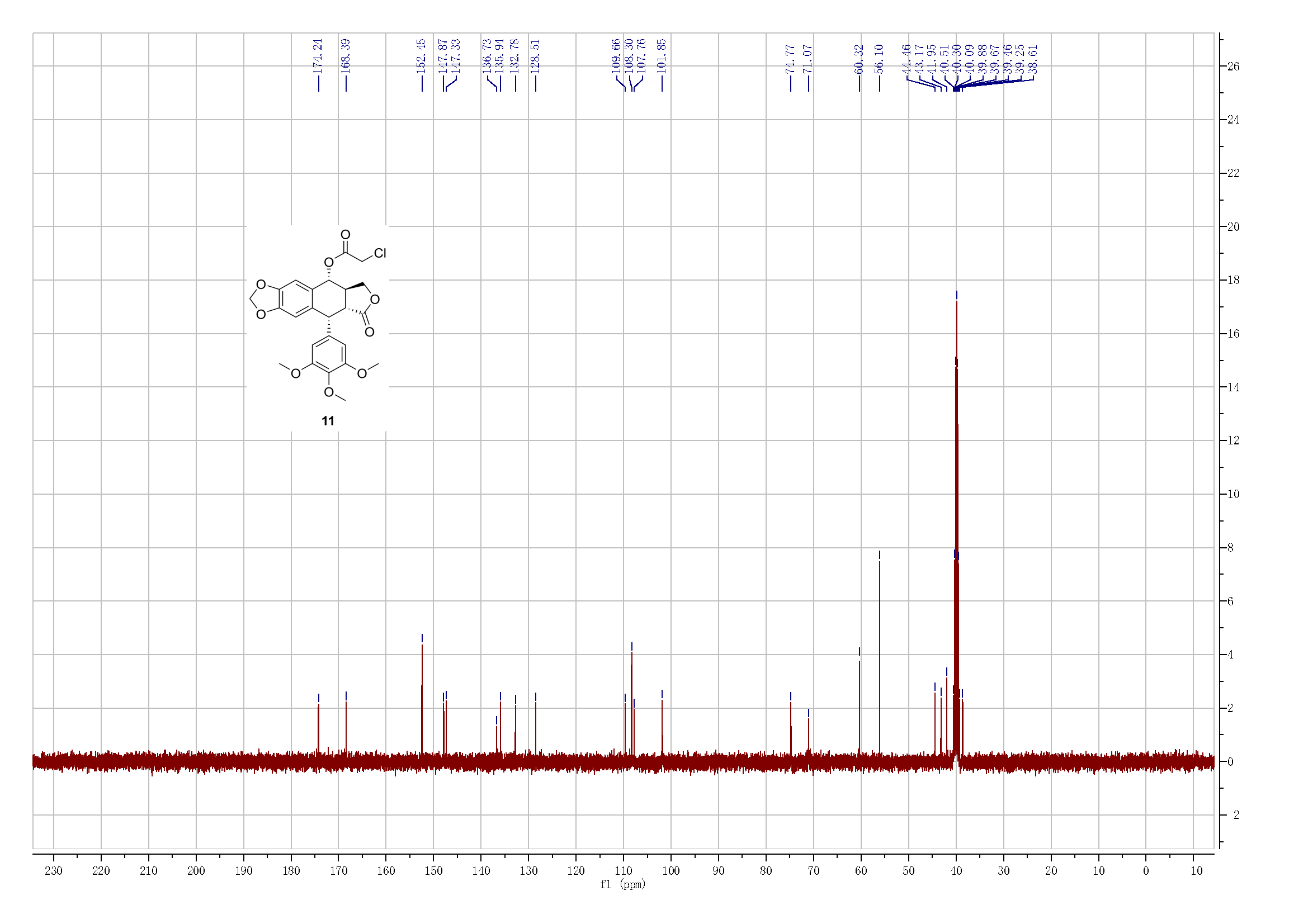


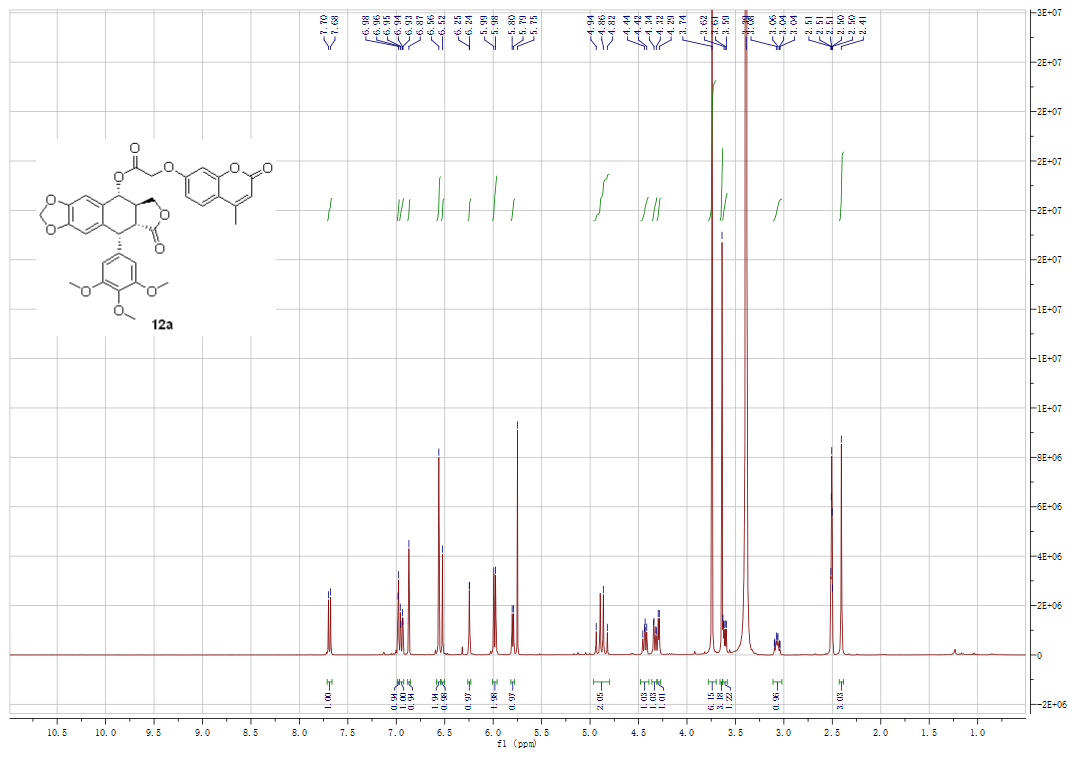


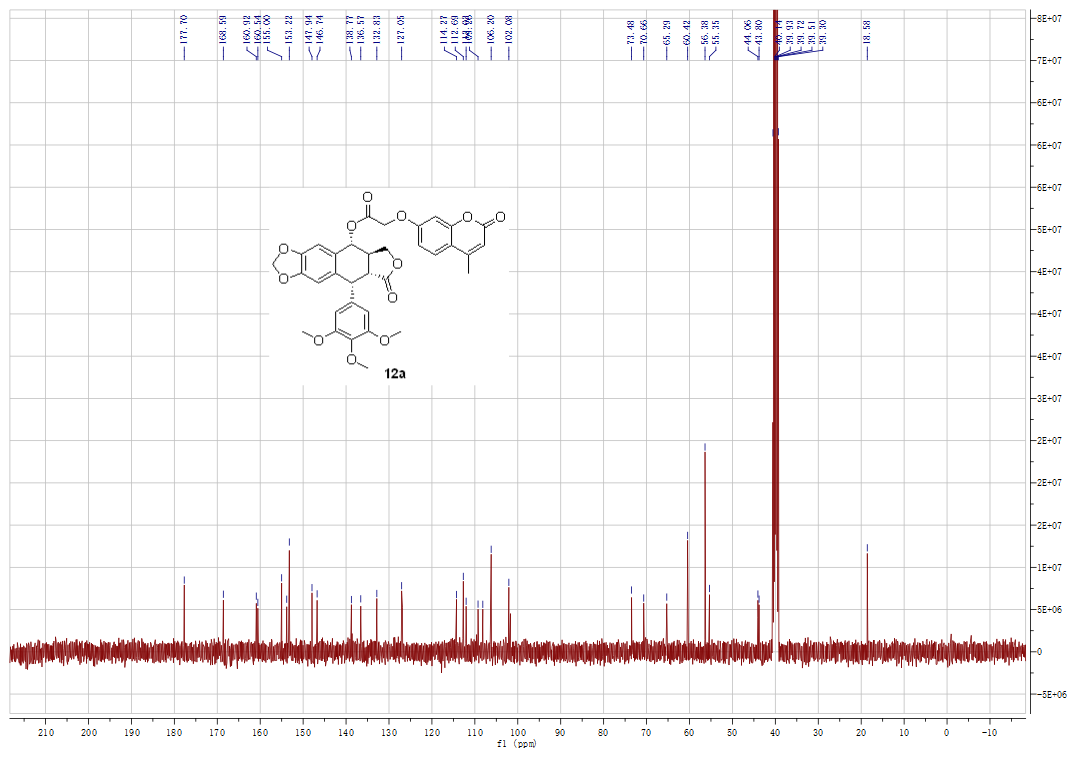


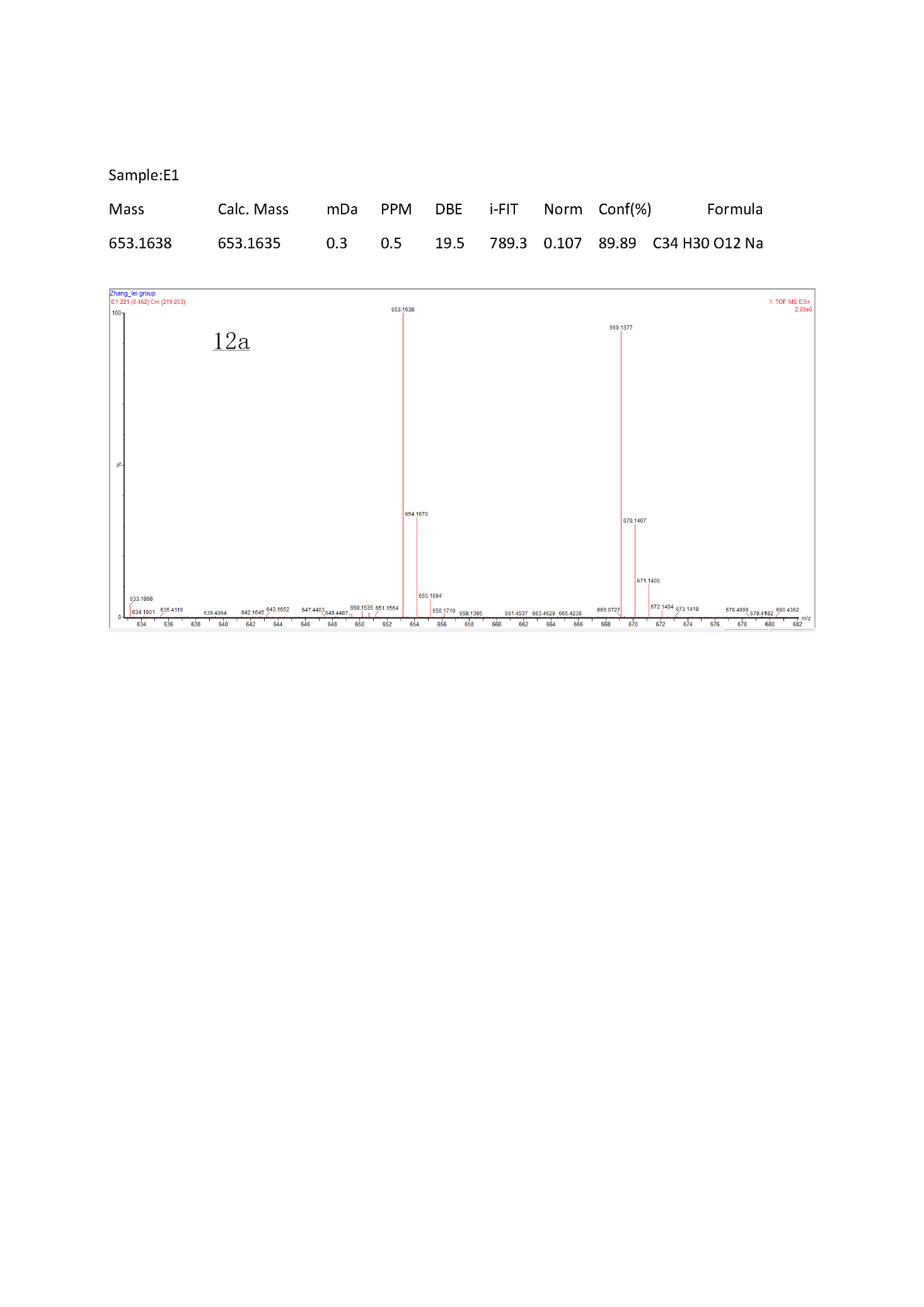


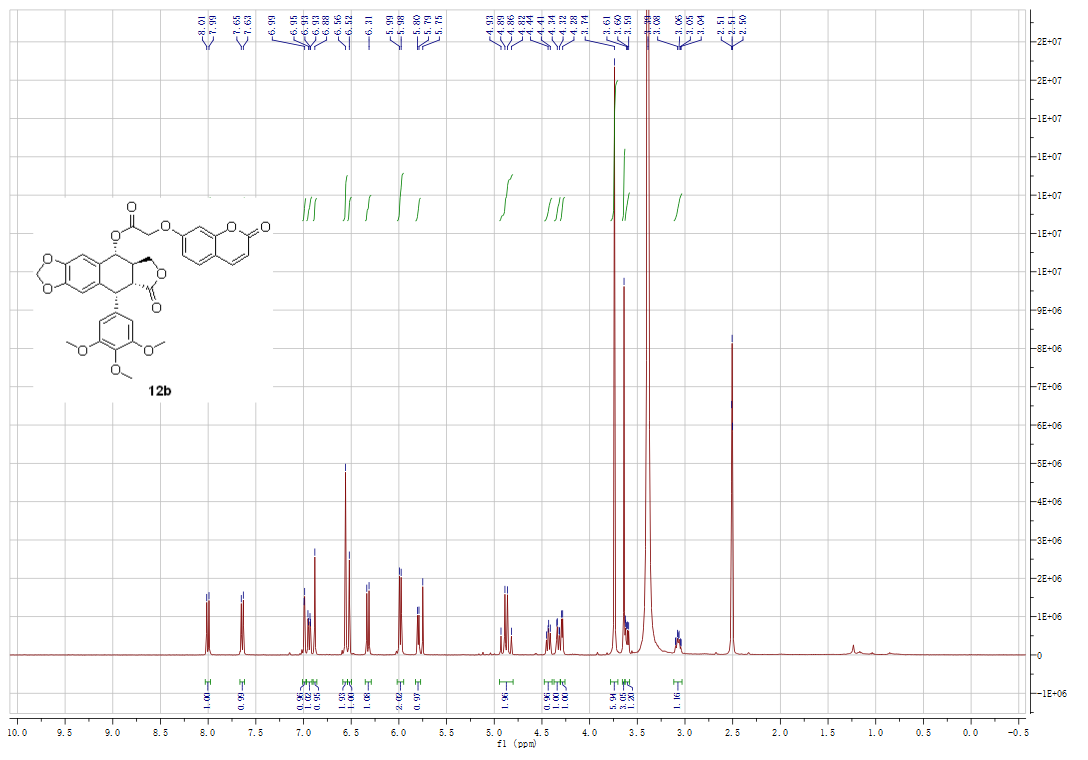


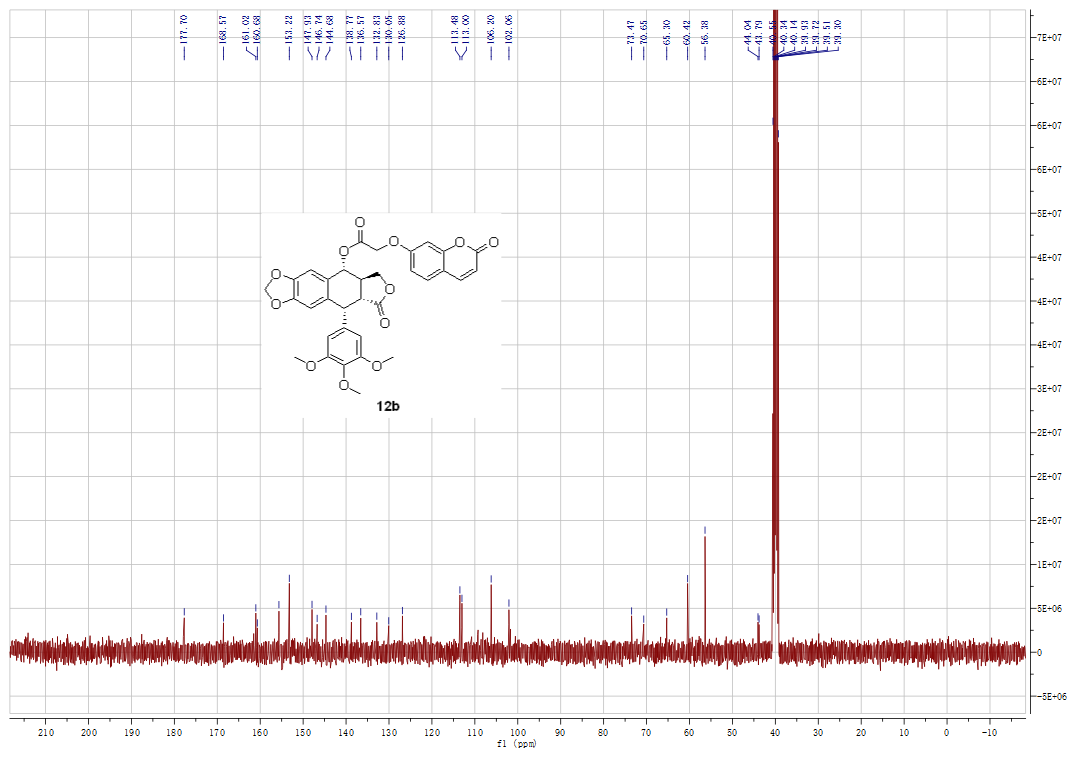


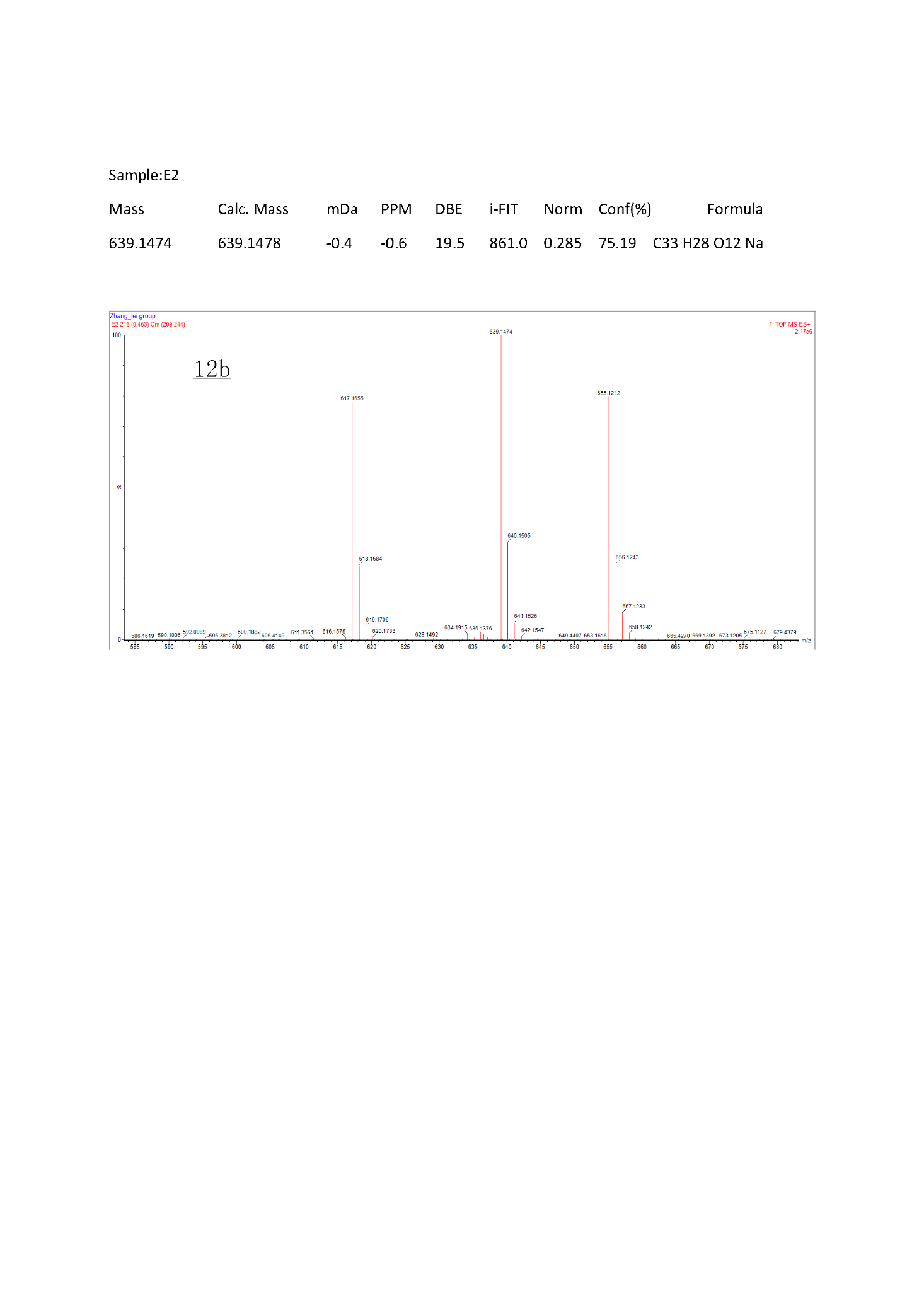


**Table S1**: Purity data of final compounds (HPLC)

| Compound | Time retention (min) | Purity % (HPLC)^a^ |
| --- | --- | --- |
| **12a** | 2.71 | 99.49 |
| **12b** | 2.53 | 99.16 |

^a^Column: WondaSIL C18-WR(4.6 mm×150 mm×5µm); Mobile phase: Acetonitrile-Water (70: 30, V/V); Wavelength: 254 nm; Rate: 1.0 mL/min; Temperature: 35°C; Injection volume: 5 μL.

**12a**


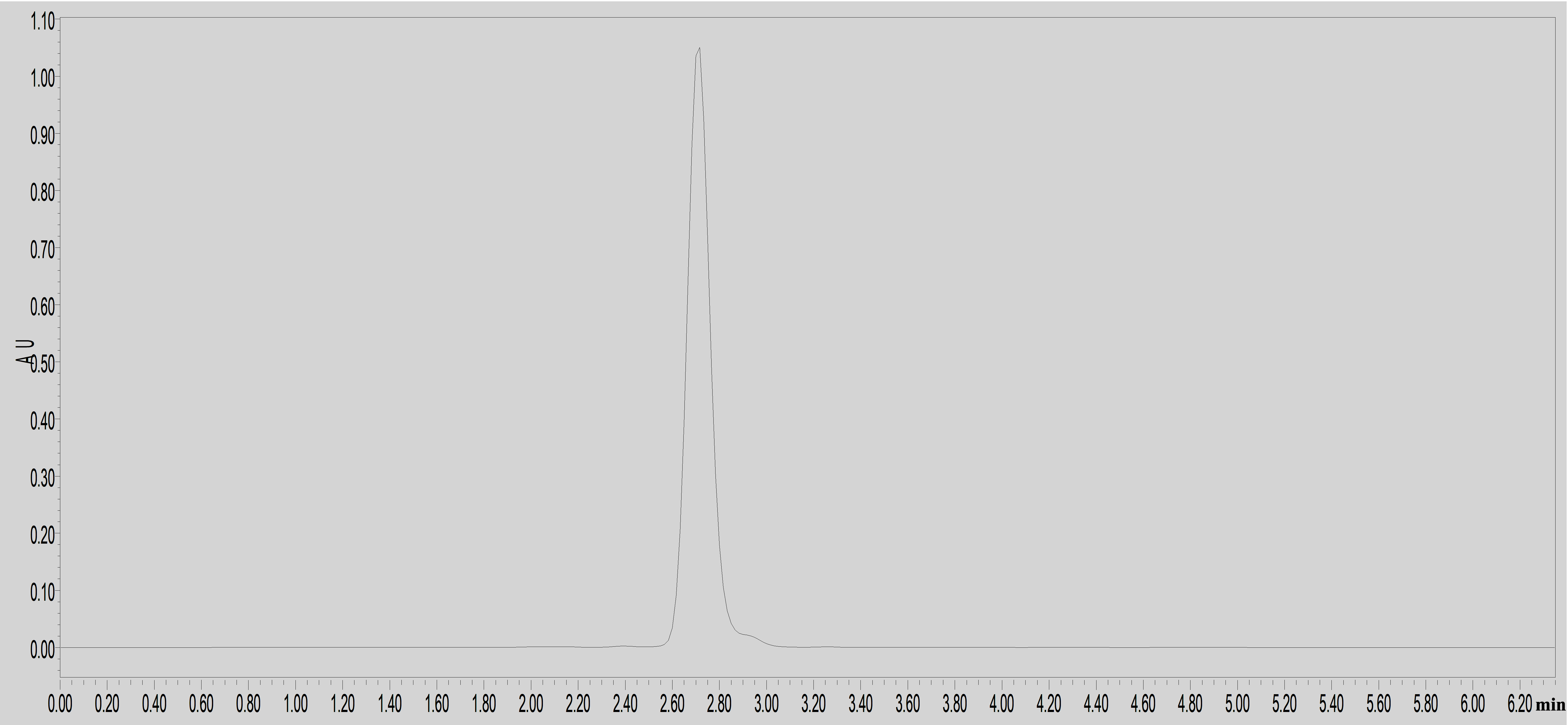


**12b**


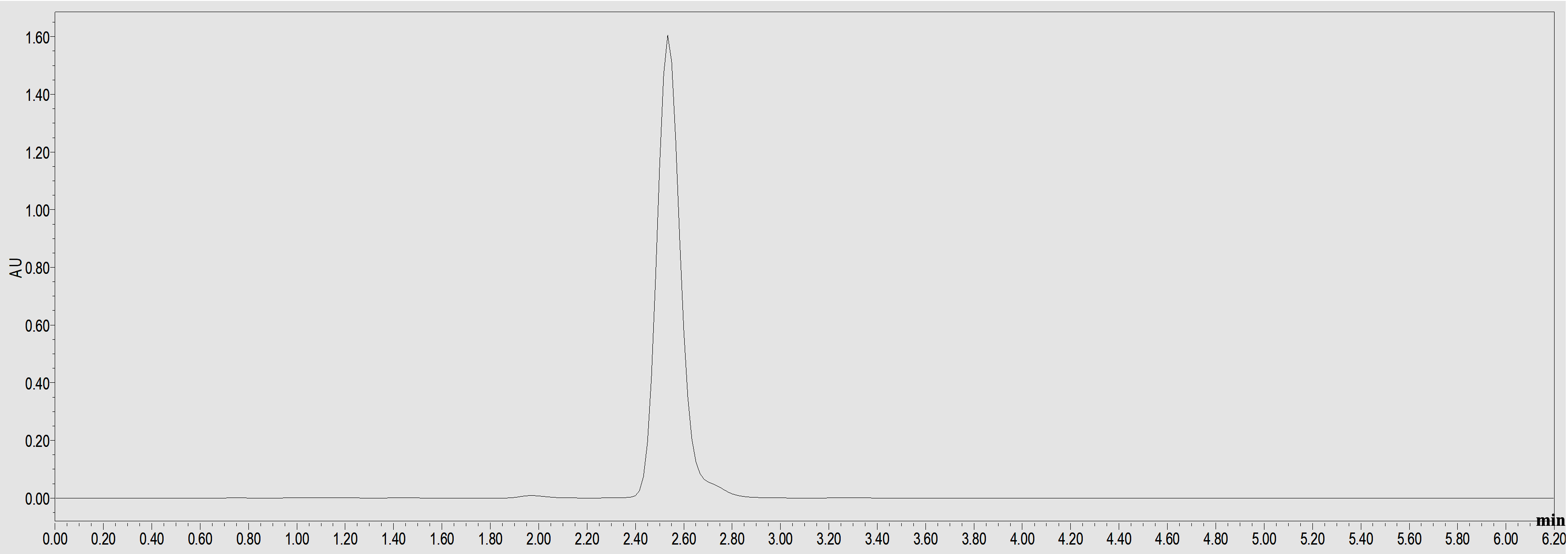

Supplement: Supplementary file 1 [file Data_Sheet_1.docx]
